# Prospective Multi-Site Validation of AI to Detect Tuberculosis and Chest X-Ray Abnormalities

**DOI:** 10.1056/aioa2400018

**Published:** 2024-09-26

**Authors:** Sahar Kazemzadeh, Atilla P. Kiraly, Zaid Nabulsi, Nsala Sanjase, Minyoi Maimbolwa, Brian Shuma, Shahar Jamshy, Christina Chen, Arnav Agharwal, Charles T. Lau, Andrew Sellergren, Daniel Golden, Jin Yu, Eric Wu, Yossi Matias, Katherine Chou, Greg S. Corrado, Shravya Shetty, Daniel Tse, Krish Eswaran, Yun Liu, Rory Pilgrim, Monde Muyoyeta, Shruthi Prabhakara

**Affiliations:** 1 Google, Mountain View, CA, USA; 2 Centre for Infectious Disease Research in Zambia (CIDRZ), Lusaka, Zambia; 3 Advanced Clinical, Deerfield, IL

## Abstract

**BACKGROUND:**

Using artificial intelligence (AI) to interpret chest X-rays (CXRs) could support accessible triage tests for active pulmonary tuberculosis (TB) in resource-constrained settings.

**METHODS:**

The performance of two cloud-based CXR AI systems — one to detect TB and the other to detect CXR abnormalities — in a population with a high TB and human immunodeficiency virus (HIV) burden was evaluated. We recruited 1978 adults who had TB symptoms, were close contacts of known TB patients, or were newly diagnosed with HIV at three clinical sites. The TB-detecting AI (TB AI) scores were converted to binary using two thresholds: a high-sensitivity threshold and an exploratory threshold designed to resemble radiologist performance. Ten radiologists reviewed images for signs of TB, blinded to the reference standard. Primary analysis measured AI detection noninferiority to radiologist performance. Secondary analysis evaluated AI detection as compared with the World Health Organization (WHO) targets (90% sensitivity, 70% specificity). Both used an absolute margin of 5%. The abnormality-detecting AI (abnormality AI) was evaluated for noninferiority to a high-sensitivity target suitable for triaging (90% sensitivity, 50% specificity).

**RESULTS:**

Of the 1910 patients analyzed, 1827 (96%) had conclusive TB status, of which 649 (36%) were HIV positive and 192 (11%) were TB positive. The TB AI’s sensitivity and specificity were 87% and 70%, respectively, at the high-sensitivity threshold and 78% and 82%, respectively, at the balanced threshold. Radiologists’ mean sensitivity was 76% and mean specificity was 82%. At the high-sensitivity threshold, the TB AI was noninferior to average radiologist sensitivity (P<0.001) but not to average radiologist specificity (P=0.99) and was higher than the WHO target for specificity but not sensitivity. At the balanced threshold, the TB AI was comparable to radiologists. The abnormality AI’s sensitivity and specificity were 97% and 79%, respectively, with both meeting the prespecified targets.

**CONCLUSIONS:**

The CXR TB AI was noninferior to radiologists for active pulmonary TB triaging in a population with a high TB and HIV burden. Neither the TB AI nor the radiologists met WHO recommendations for sensitivity in the study population. AI can also be used to detect other CXR abnormalities in the same population.

## Introduction

Globally, 30 to 40% of people with tuberculosis (TB) are undiagnosed, with much of this burden concentrated in low-to-middle-income countries (LMICs).^1^ A lack of diagnosis contributes to worse outcomes and disease transmission, hampering progress toward the End TB Strategy goals of the World Health Organization (WHO).^2^ A major factor contributing to the missed or delayed diagnosis of TB is limited access to and cost of highly sensitive, easy-to-use screening tools.^3^ The most widely available screening tool is symptom screening, which lacks sensitivity, specificity, or both.^4^ In many settings, chest radiography, or chest X-ray (CXR), for TB-associated findings can improve early TB detection and enable prioritization for highly accurate but more costly molecular tests for confirmation. However, in many LMICs with a high TB burden, CXR use is hindered by a lack of human experts to interpret the images and by substantial intra- and interreader variability in accuracy of detecting TB.^4^

In 2021, the WHO recommended the use of computer-aided detection (CAD) software to automate digital CXR readings as an alternative to human interpretation, with target thresholds for these products of 90% sensitivity and 70% specificity.^5^ Prospective validation studies are needed, as both human TB-screening performance and CAD-based TB-screening performance differ based on factors such as human immunodeficiency virus (HIV) prevalence, prior TB treatment, and sex.^6^ In addition, CAD systems for TB detection that fail to consider other pathologies (non-TB incidental findings) may be limited in their utility as broader screening tools.^7^

Previous studies have focused on predominantly HIV-negative populations, or did not have data on HIV prevalence.^8–10^ Two commercially available TB CAD systems were retrospectively evaluated and shown to have met the WHO-recommended target profile.^8^ A recent abnormality-detecting AI (abnormality AI) study focused on a patient population over 65 years of age and achieved 99.8% sensitivity and 36% specificity, suggesting the ability to prioritize CXRs for reads and reduce workload.^9^ In another study, in Finland, an abnormality AI achieved 98% sensitivity and 33% specificity.^10^ However, patients with HIV coinfected with TB often have an atypical CXR presentation, making TB screening more complex and indicating a need for prospective data on the performance of AI systems on populations with a high HIV burden.^11–13^

We conducted a prospective study evaluating AI for TB detection (TB AI) in a setting with a high TB and HIV burden. We also employed a second AI, an abnormality AI, that detects general CXR abnormalities to understand the feasibility of using AI to aid the detection of other abnormalities during TB screening.

## Methods

The study was conducted between November 2021 and February 2023 in three health facilities in the Lusaka district of Zambia (Chawama, Kanyama, and Chainda), all of which provide TB diagnostic and treatment services. All participants provided written informed consent. The study had ethics approval from the University of Zambia Biomedical Research Ethics Committee (#1989–2021) and was preregistered on clinicaltrials.gov (NCT05139940).

**Figure 1.**
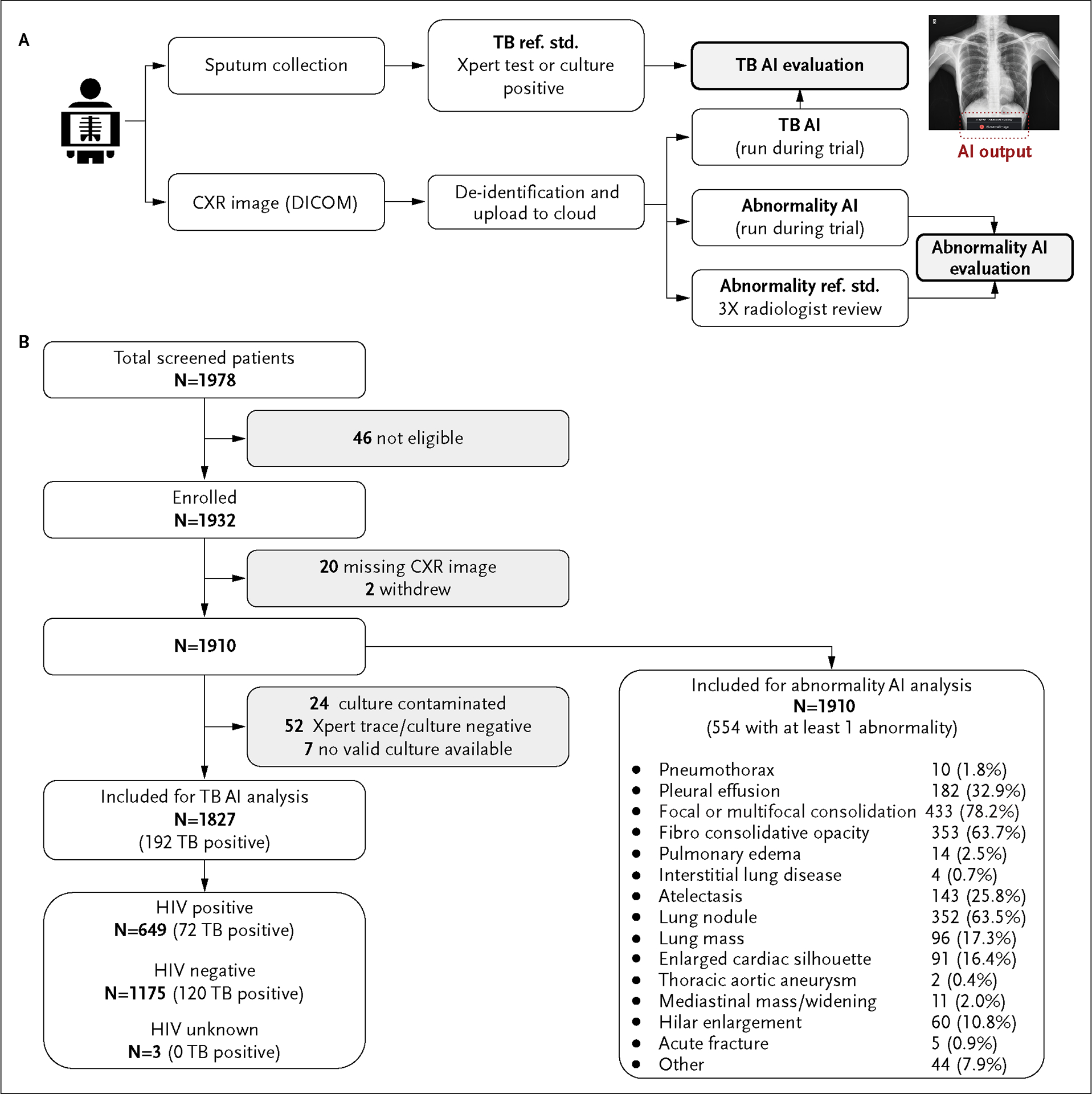
Prospective Observational Study Overflow and Patient Flow. A study participant undergoes CXR imaging and samples are sent for GeneXpert testing and culture. The de-identified CXR is uploaded into a cloud-based DICOM store and two AI models are run during the study (TB and abnormality) as beam pipelines, and the results are written within 24 hours of upload. Note that, as an observational study, the AI outputs did not influence patient care (Panel A). STARD diagram illustrating patient flow and exclusions (Panel B). CXR denotes chest X-ray; DICOM, Digital Imaging and Communications in Medicine; Ref. Std., reference standard; and TB, tuberculosis.

Patients who were 18 years of age or older and who met any of the following three conditions were included: presumptive TB patient, regardless of HIV status, namely presentation with any of the four cardinal symptoms of TB (cough, fever, weight loss, or night sweats); household contact with a TB patient; and a newly diagnosed HIV patient. Persons on current treatment or within 1 year of treatment for active TB were not included. Eligible patients underwent digital CXR, clinical evaluation (medical history and physical examination), and sputum sample collection. Digital CXRs were de-identified and uploaded to a Google Cloud Digital Imaging and Communications in Medicine (DICOM) store using the DICOMWeb application programming interface. Both AI systems (TB and abnormality) were run daily on all uploaded images through two Apache Beam pipelines that collected the DICOMs, ran inference, and then wrote results into new DICOM images, which were saved in a second set of DICOM stores. AI results were not available to the study team during the visit and did not influence patient care. Figure 1A summarizes the study process. Details about the database, data validation, and data access are provided in Supplementary Appendix.

### AI MODELS (TB AND ABNORMALITY) AND PRESELECTED THRESHOLDS

Both AI models accepted a single frontal CXR DICOM image as input for each participant. The training data and validation of earlier versions of both AI models were described previously and both were updated prior to study commencement (see Supplementary Appendix for details).^6,14^ Each model produced a score between 0 and 1, and scores were converted to binary (positive or negative) using prespecified thresholds derived from a pilot phase of 493 patients preceding the main study (see AI Model Threshold Selection Process in the Supplementary Appendix). Pilot data are not used for the statistical analysis presented here.

### MICROBIOLOGICAL TESTING AND REFERENCE STANDARD

To determine the study participants’ TB status, sputum samples were sent for Xpert Ultra testing (Cepheid, Sunnyvale, CA, USA), smear, and *Mycobacterium* culture at the Centre for Infectious Disease Research in Zambia (CIDRZ) central laboratory. An extra sample was sent to the local laboratory for Xpert Ultra testing. At the central laboratory, samples were prepared using standard operating procedures for TB testing. Samples were processed using the NALC-NaOH method and the remaining pellet at the end of the decontamination process was used to prepare a smear, to inoculate onto solid Löwenstein–Jensen media and liquid mycobacteria growth indicator tube (MGIT) media, and to test with Xpert Ultra. Positive culture samples were identified using Ziehl–Neelsen staining followed by a BD MGIT TBc identification test for acid-fast bacilli-positive cultures, to confirm the presence of *Mycobacterium tuberculosis*. The reference standard for TB was defined as *Mycobacteria tuberculosis* isolated on culture or detected at very low to high levels through Xpert Ultra *Mtb* testing. Xpert Ultra “trace” results were included only if the culture was positive.

The reference standard for CXR abnormality presence was the majority vote of three India-based radiologists, selected from a pool of 19 based on availability and experience, and had 8, 12, and 14 years of experience reviewing CXRs for suspected TB. Radiologists underwent onboarding to become familiarized with the question template for image labeling. They then reviewed each image independently and provided a binary (yes/no) answer for the presence of potentially actionable findings that could be considered abnormal, the type of abnormality, and whether there were any minor technical issues visible on each image. The type of abnormality (Fig. 1), was defined by the union of the indicated findings.

### COMPARATOR RADIOLOGIST READS: TB AND ABNORMALITY AI

To contextualize TB AI performance, 10 India-based radiologists (mean years of experience, 9.1±2.0; range, 6–12) labeled each image for active TB status. To standardize reads, all radiologists were instructed, “Assume this CXR is taken at a TB screening center in a TB endemic region; please read for high sensitivity to TB.” As a comparison for the abnormality AI’s performance, an additional six India-based radiologists (mean years of experience, 8.8±2.6; range, 6–12) reviewed the images using the same instructions as those used for the reference standard radiologists (presence and type of abnormality). All radiologist reads were performed without knowledge of the clinical information, since the clinical data were not available for use at the time of image review.

### STATISTICAL ANALYSIS

All hypothesis tests described in this section were prespecified unless indicated. The primary analysis compared the sensitivity and the specificity of the TB AI with those of radiologists, using a 5% noninferiority margin and a one-sided alpha of 2.5% (after multiple testing correction for two tests). Superiority testing was performed if noninferiority was met, which does not require multiple testing correction.^15^ Consistent with prior work,^6^ we used the Obuchowski–Rockette–Hillis procedure^16,17^ configured for binary data^18^ and adapted it to compare readers with the standalone algorithm^19^ in a noninferiority setting.^20^

Secondary analyses used the same methodology for evaluating TB AI’s noninferiority to the WHO’s targets of 90% sensitivity and 70% specificity, and the abnormality AI’s noninferiority to 90% sensitivity and 50% specificity. The abnormality AI’s target was chosen to favor sensitivity over specificity based on an estimate of useful performance in a triage system for image review.^10^ Exploratory analyses included subgroup analyses based on clinical site, HIV status, age, sex, sputum smear status, CXR quality, and the presence of foreign objects. For the TB AI, additional analyses assessed specificity at sensitivities matching the mean radiologist sensitivity and at 90% sensitivity. No images had foreign objects indicated upon review, so this subgroup analysis was skipped. All 95% confidence intervals were calculated using bootstrapping across cases, and, where relevant, also across raters.

Each of the AI models had two thresholds prespecified. For the TB AI model, a high-sensitivity threshold (0.305) was selected to favor sensitivity over specificity given the WHO targets, and a balanced exploratory threshold (0.465) was selected to approximate radiologist performance. The respective thresholds for the abnormality AI were 0.54 and 0.67.

## Results

A total of 1932 participants were enrolled, of whom 20 (1.0%) were missing a digital CXR and 2 (0.1%) withdrew. Data from all remaining participants were used to evaluate the abnormality AI. For the TB AI analysis, another 83 (4.3%) were excluded due to lack of reference standard: contaminated samples (24), trace results on Xpert testing without culture positivity (52), or invalid Xpert results (7) (Fig. 1). Of the 1827 included in the final TB analysis, 975 (53.4%) were female, the median age was 35 years (interquartile range, 27–45), 649 (35.5%) had HIV-positive status, 301 (16.5%) reported previous TB treatment and 1082 (59.2%) had three or more symptoms (Table 1). Based on microbiological or molecular testing (see “Methods” section), 192 (10.5%) had TB.

### TB AI PERFORMANCE

**Figure 2.**
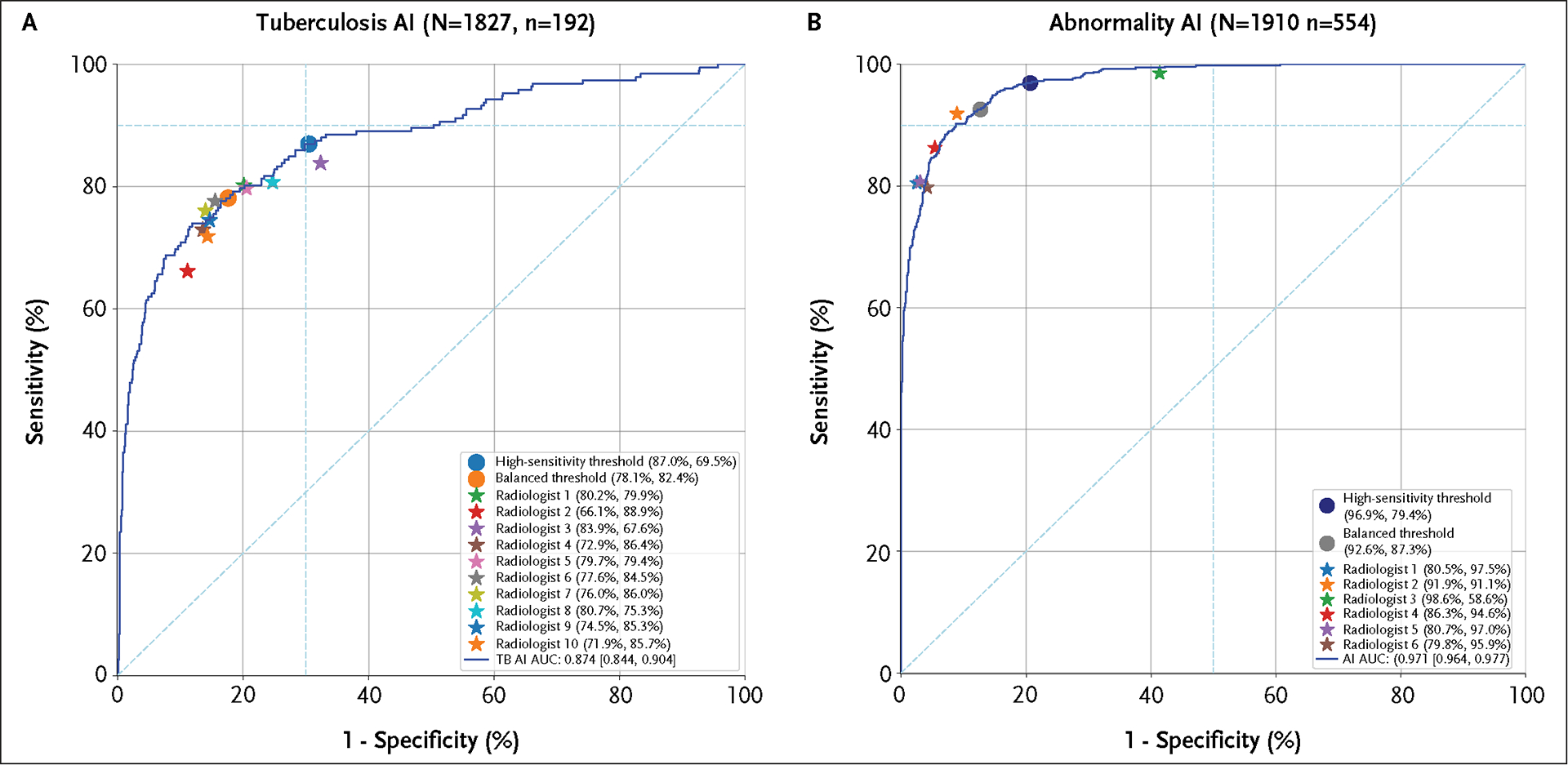
Performance of the Tuberculosis AI and Abnormality AI. Panel A shows the performance of the TB AI and Panel B shows the performance of the abnormality AI. Radiologist performance is plotted as stars alongside the AI’s receiver operating characteristic curves. “N” represents patient count, while “n” represents TB-positive patient count (panel A) and abnormality-positive patient count (panel B). Sensitivity and specificity values at AI model thresholds and radiologist performance are indicated in parentheses, and 95% CIs for the AUC are indicated in square brackets. The TB AI had two thresholds, with the high-sensitivity threshold meeting WHO targets for specificity but not sensitivity, and the balanced threshold being noninferior to radiologists for both sensitivity and specificity. The abnormality AI system similarly had two thresholds, with the high-sensitivity threshold meeting the prespecified targets of 90% sensitivity and 50% specificity. AUC denotes the area under the receiver operating characteristic curve; CI, confidence interval; TB, tuberculosis; and WHO, World Health Organization.

The TB AI had an area under the receiver operating characteristic curve (AUC) of 0.87 (95% confidence interval [CI], 0.84 to 0.90) (Fig. 2A). To compare the performance of the AI model to that of human readers, 10 radiologists reviewed the images, achieving a mean sensitivity of 76% (95% CI, 71 to 83) and mean specificity of 82% (95% CI, 77 to 85). In the primary analysis, at the high-sensitivity threshold preselected to resemble the WHO target profile, the TB AI’s sensitivity (87% [95% CI, 82 to 92]) was higher than that of the India-based radiologists (difference, 11% [95% CI, 5.6 to 15]; P<0.001 for AI superiority), but the TB AI’s specificity (70% [95% CI, 67 to 72]) was not (difference, −12% [95% CI, −16 to −7.2]; P=1 for AI noninferiority). At the exploratory balanced threshold, both the TB AI’s sensitivity (78% [95% CI, 72 to 84]) and specificity (82% [95% CI, 81 to 84]) were comparable (noninferior) to those of the radiologists (sensitivity difference, 1.8% [95% CI, −3.3 to 6.4]); specificity difference, 0.48% [95% CI, −2.9 to 5.2]).

In comparison with the WHO targets of 90% sensitivity and 70% specificity, the AI model at the high-sensitivity threshold met the specificity target, at 70%, but was under the sensitivity target, at 87%. None of the radiologists’ individual point estimates met the WHO targets for sensitivity, although all met the specificity targets. The highest sensitivity achieved by a radiologist was 84% and that radiologist’s specificity was 68%.

In exploratory analyses matching to average radiologist performance, the TB AI had a 1.2% higher specificity than radiologists when matching the mean radiologist sensitivity of 76%, and a 2.1% higher sensitivity when matching the mean radiologist specificity of 83%. Exploratory analyses comparing the TB AI to each individual radiologist and WHO targets are presented in Tables S1–S3.

#### Subgroups

**Figure 3.**
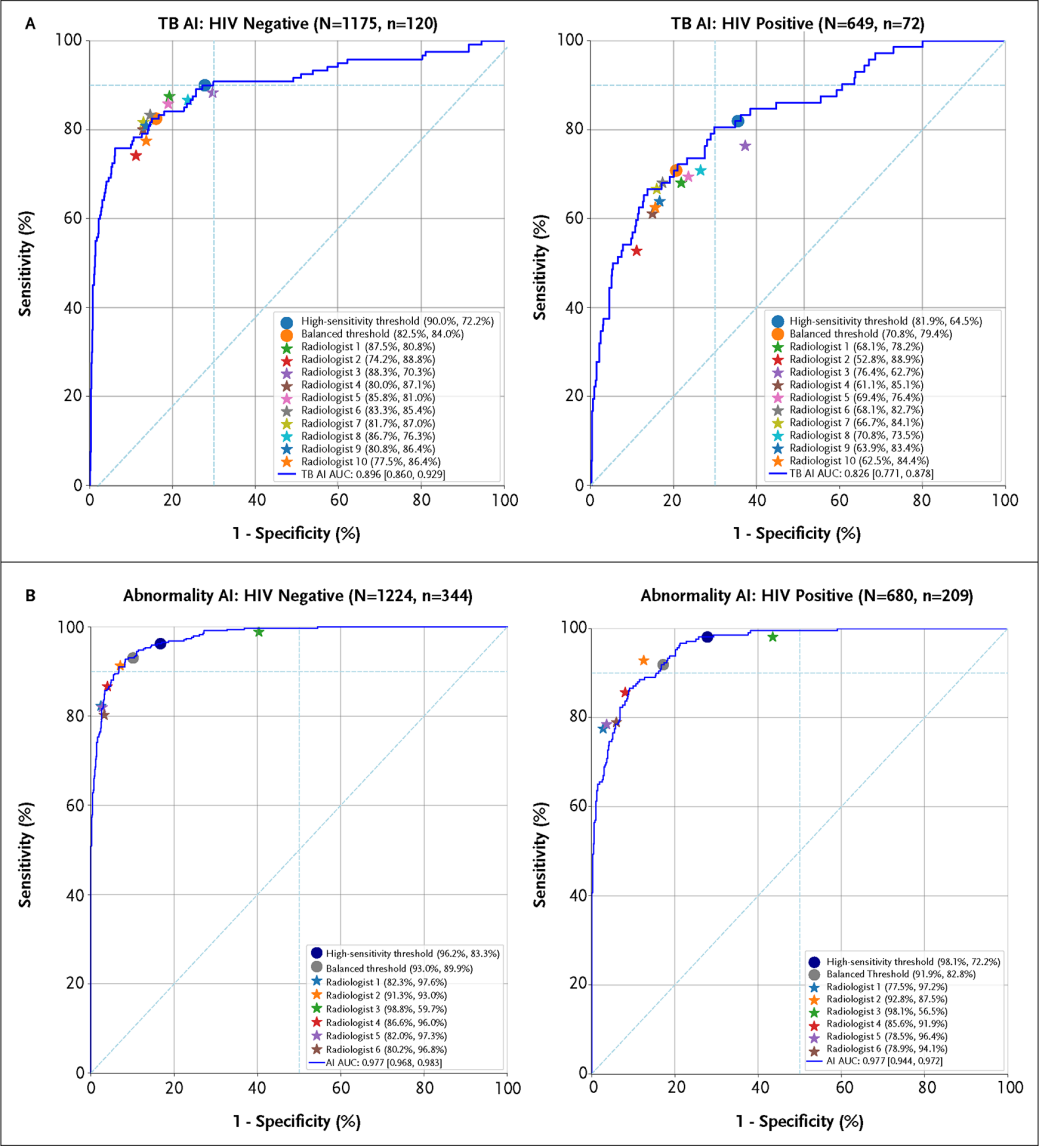
Subgroup Analysis of the Tuberculosis AI and Abnormality AI by HIV Status. Panel A shows the performance of the TB AI and Panel B shows the performance of the abnormality AI. AUC denotes the area under the receiver operating characteristic curve; and TB, tuberculosis.

We next considered subgroups based on HIV status, previous TB status, age, and sex. Overall, AI and radiologist performances tended to vary in similar ways across subgroups. When stratified by HIV status (Fig. 3A), the TB AI had an AUC of 0.90 (95% CI, 0.86 to 0.93) in the HIV-negative subgroup and an AUC of 0.83 (95% CI, 0.77 to 0.88) in the HIV-positive subgroup. The TB AI’s sensitivity met the WHO targets (90% sensitivity, 72% specificity) in the HIV-negative subgroup, but not in the HIV-positive subgroup (82% sensitivity, 65% specificity). Similarly, radiologists had lower sensitivity and specificity for the HIV-positive group than for the HIV-negative group (mean sensitivity: 66% vs. 83%, mean specificity 80% vs. 83% for HIV-positive and HIV-negative subgroups, respectively). In terms of previous TB status (Fig. S1), the TB AI had an AUC of 0.89 (95% CI, 0.86 to 0.93) for those without a prior TB diagnosis and an AUC of 0.76 (95% CI, 0.66 to 0.85) for those with a prior TB diagnosis. While radiologist sensitivity remained similar, radiologist specificity decreased substantially for patients with a prior TB diagnosis.

For performance stratified by sex, both the AI and radiologists had similar differences in performance, with lower sensitivity and higher specificity for female patients (5% TB positive) than for male patients (17% TB positive). In the female subgroup, the TB AI had an AUC of 0.84 (95% CI, 0.77 to 0.91), while, for the male subgroup, the AUC was similar, at 0.86 (95% CI, 0.83 to 0.90), indicating that the shift was most prominent in the threshold predictions (Fig. S2A). Evaluating performance by age, the TB AI AUC remained near 0.90 for all but the 40-to-65-year-old group, where the AUC was 0.81 (95% CI, 0.74 to 0.87). Similarly, radiologists’ sensitivity declined for this group (Fig. S3A). For detailed subgroup analyses, see Supplementary Appendix and Figures S1–S4 (Panel A of each figure) and Figure S5.

### ABNORMALITY AI RESULTS

The abnormality AI was evaluated on a broader set of cases than the TB AI because the reference standard relies on radiologist CXR reviews; indeterminate results of the TB reference standard (e.g., trace Xpert testing, missing or contaminated cultures) did not require exclusions. For 1910 cases (554 with abnormalities [29%]), the abnormality AI achieved an AUC of 0.97 (95% CI, 0.96 to 0.98). The high-sensitivity threshold achieved a sensitivity of 97% (95% CI, 95 to 98) and a specificity of 79% (95% CI, 77 to 81), meeting the secondary statistical end points of 90% sensitivity and 50% specificity. The exploratory balanced threshold achieved a sensitivity of 93% (95% CI, 90 to 95) for meeting the same end point and a specificity of 87% (95% CI, 85 to 89). These results are plotted along with the comparative performance of six radiologists in Figure 2B.

In exploratory analyses, relative to the six comparator radiologists, the abnormality AI at the high-sensitivity threshold achieved an 11% higher sensitivity and a 10% lower specificity. The balanced threshold resulted in a 6% higher sensitivity and a 2% lower specificity than the radiologists.

Subgroup analyses are shown in Figure 3B and Figures S1–S4 (Panel B of each figure). Performance shifts for the abnormality AI in the subgroups resembled that of the TB AI, albeit with smaller effect sizes. For example, in the “previous TB” and “HIV-positive” subgroups both the abnormality AI and radiologists had lower specificity. In the case of the sex subgroup analysis, both sensitivity and specificity were higher for male patients. Specificity decreased with increasing age, while sensitivity started to increase for the subgroups of participants greater than 40 years of age. Across the subgroup analyses, the high-sensitivity threshold was more sensitive than five of the six radiologists, although the results were not statistically significant. Further supplementary analysis is provided in the Supplementary Appendix.

### QUALITATIVE IMAGE REVIEW

**Figure 4.**
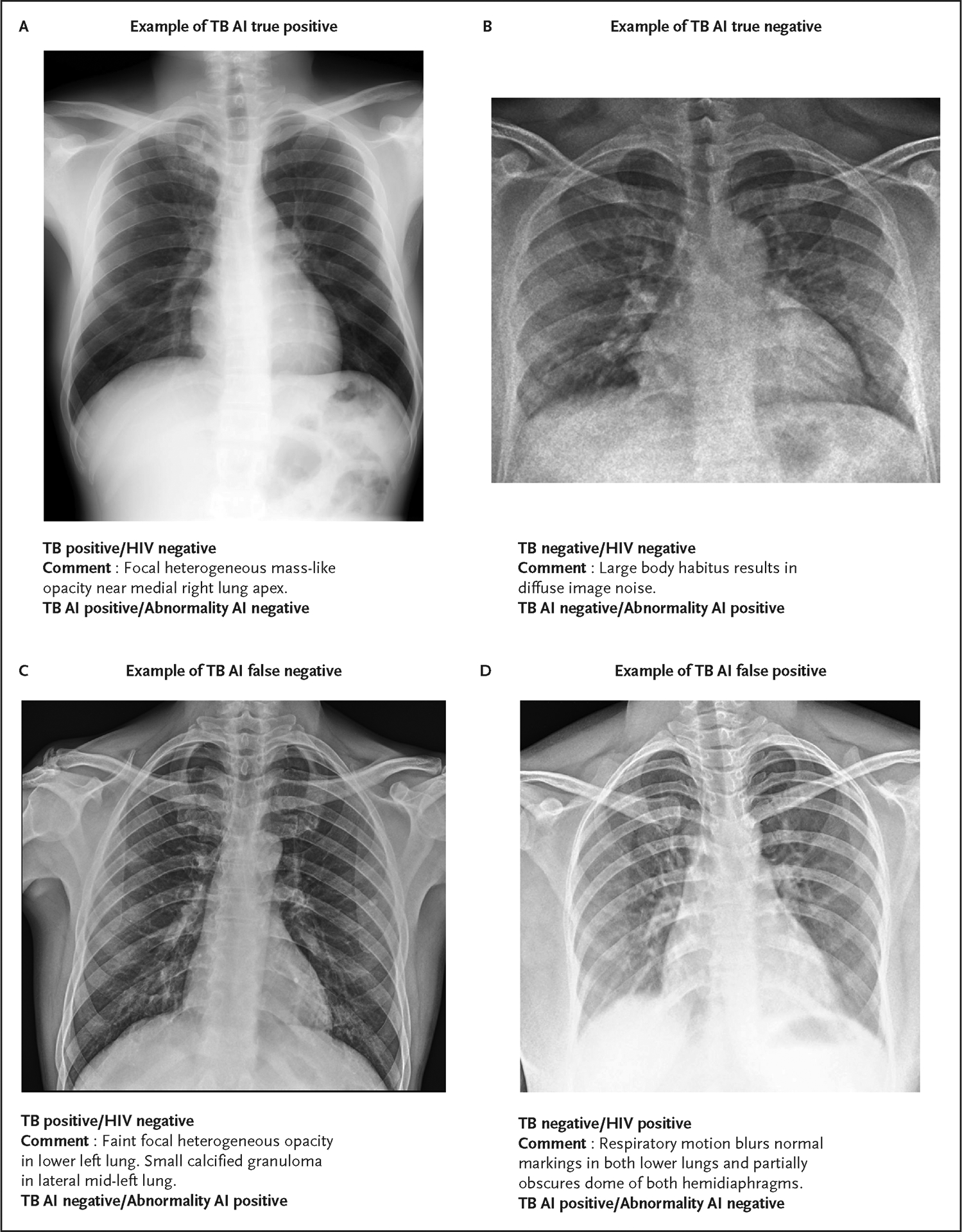
Example Chest X-Rays on Borderline Decision Categories and AI Model Differences. Panel A shows true positive cases where the abnormality AI model is deemed negative, Panel B shows an example of a TB true negative where the abnormality AI was positive, Panel C shows a TB AI false negative with an abnormality AI positive, and Panel D shows a TB AI false positive with an abnormality AI negative. Comments per panel reflect those from an experienced thoracic radiologist. TB denotes tuberculosis.

Cases of model and/or reader disagreement were randomly selected (24 total) and qualitatively reviewed by an experienced thoracic radiologist. These cases were divided into TB AI true and false positives and negatives where either the readers or the abnormality AI disagreed. Overall, TB AI false negatives were found to appear normal with mild scarring or calcified granulomas. TB AI false positives were also found to appear normal but some contained different imaging artifacts or interference (e.g., motion blurring, faint opacification due to breast tissue). A subset of these cases is shown in Figure 4. Additional discussion is presented in Supplementary Appendix.

## Discussion

The TB AI’s performance was comparable to radiologists overall but its behavior differed in potentially clinically meaningful ways; for example, the AI model at the high-sensitivity threshold was more sensitive and less specific than the radiologists. The abnormality AI met the prespecified targets, performing well despite considerable conflation with chest infections in the COVID-19 era and in a population with a high HIV burden, which suggests that the AI model may generalize in similarly challenging environments.

In 2021, the incidence of TB in Zambia was 307 per 100,000 individuals.^21^ The high prevalence of HIV in our study population enabled subanalyses of AI with respect to the WHO’s target product profile in this population.^22^ While radiologist sensitivity was 70 to 90% in the HIV-negative subgroup, this dropped to 50 to 70% in the HIV-positive subgroup. Since the CXRs of HIV-positive patients with TB are more likely to exhibit nonclassical features or appear normal, this was expected. As such, while some radiologists were closer to the WHO targets in the HIV-negative population, none met the WHO targets for patients with HIV. The TB AI’s performance trended the same way, with an 8% absolute reduction in sensitivity in HIV-positive versus HIV-negative patients. The relative performance of the AI model and radiologists for HIV-positive patients suggests that AI could be particularly useful for detecting TB in a population with HIV. However, more research is needed to understand whether the same WHO targets are feasible for detecting TB in patients with HIV.

The AI’s performance was comparable to that of the radiologists across most subgroups. However, variations in performance highlight the influence of patient population characteristics on both radiologist and AI performance, suggesting the need for population-level tuning of AI thresholds, methods for which are described by the StopTB Partnership.^23^ Performance shifts for both radiologists and AI based on previous TB status and sex were previously reported in retrospective studies.^7^ For age, however, the poorer performance for radiologists and AI in 40-to-65-year-old patients than in younger patients was initially surprising, but this trend is likely to be due to the correlation of HIV status and prior TB status with age. The trend weakened when the age subgroup analysis was further stratified to HIV-negative participants without prior TB (Supplementary Results).

Since symptoms that present as TB may be caused by non-TB infection or malignancy, concurrent CXR abnormality screening can provide more comprehensive care. Similarly to published work, the abnormality AI prespecified targets of 90% sensitivity and 50% specificity were selected to optimize for triaging use cases, where sensitivity is more important than specificity.^10^

Although digital CXR acquisition may be less than one tenth of the cost of an on-site GeneXpert test,^24^ the use of CXR for TB screening is bottlenecked by the access and cost of experts for CXR interpretation.^25^ In addition, the AI model can be tuned to local populations, to potentially provide better performance than teleradiologists less familiar with the local population. Moreover, because the AI models’ output is a continuous score between 0 and 1 (before thresholding), these thresholds can be calibrated to meet site-specific resource constraints and practice patterns, which can be adjusted as the local prevalence of TB changes over time.^23^

Prior evidence suggests that AI for TB screening may help reduce delays in diagnosis and care. A meta-analysis of delays in TB diagnosis across 78 countries showed a mean delay of 29.5 days from the first visit to a qualified facility until diagnosis of TB.^26^ Unlike central or teleradiologist reviews, AI can be run on demand either on premises on a single central processing unit (CPU) or graphics processing unit (GPU) or on the cloud. With inference time under a minute for each image, AI can provide access to CXR reads where experts are scarce or unavailable and thus sizably reduce the diagnosis delay. The availability of an on-demand CXR read may also help reduce costs by enabling more efficient use of molecular testing to confirm positive CXR results,^6^ thus helping to scale up screening to more patients, although additional research is needed to confirm this.

This study has several limitations. First, given that radiologist performance was incongruent with WHO targets (radiologists were more specific but less sensitive), a single AI threshold could not meet both end points. Although this study prespecified and evaluated two thresholds, further investigation and likely country- or institution-specific customization may be needed to better select the most appropriate thresholds to use in each setting.^23^ Second, after study commencement, a shared digital CXR machine for two of the sites (Kanyama and Chawama) was found to produce small grid-like artifacts in a repeating pattern due to a defect with the device (Fig. S10). This device was used for CXR at both sites, and, while the artifacts can be easily ignored by humans, every pixel was interpreted by the AI models. Because this regular repeating pattern is not known to be present in any of the training datasets, this may have resulted in reduced AI performance. The number of positive examples in the third site (Chainda) and other site-level differences (e.g., TB positivity and abnormality rates) make it hard to confidently infer any performance differences or attribute any differences to this versus other factors. Although broader impact analysis of this and other potential real-world artifacts is a subject for future research, it is encouraging that the AI models performed well nonetheless.

In summary, this prospective multisite study of a population with a high HIV burden showed that the TB AI model operating at a prespecified high-sensitivity threshold was more sensitive and less specific than radiologists. A prespecified balanced TB AI threshold was noninferior to 10 highly experienced radiologists on both sensitivity and specificity. The abnormality AI met prespecified targets. With an AI model run time of less than a minute and accuracy comparable to radiologists, this system has the potential to contribute to reducing delays in TB diagnosis and may help both enable TB screening in areas with a scarcity of radiologists and triage patients with non-TB lung conditions.

## Supplementary Material

data sharing statement

disclosures

appendix

## Figures and Tables

**Table 1.** Patient Characteristics for the Tuberculosis AI Model Study.*

Variable	All	No TB	TB Positive
Total (N, %)	1827 (100)	1635 (89.5)	192 (10.5)
Site (N, %)			
Facility 1 (Kanyama)	862 (47.2)	732 (44.8)	130 (67.7)
Facility 2 (Chawama)	742 (40.6)	689 (42.1)	52 (27.1)
Facility 3 (Chainda)	224 (12.3)	214 (13.1)	10 (5.2)
Sex (N, %)			
Female	975 (53.4)	924 (56.5)	51 (26.6)
Male	852 (46.7)	711 (43.5)	141 (73.4)
Median age (years, IQR)	35 (27–45)	35 (27–45)	34 (27–43)
HIV status (N, %)			
Positive	649 (35.5)	577 (35.3)	72 (37.5)
Negative	1175 (64.3)	1055 (64.5)	120 (62.5)
Missing	3 (0.2)	3 (0.2)	0
Previous TB treatment (N, %)			
Yes	301 (16.5)	267 (16.4)	34 (17.8)
No	1522 (83.3)	1364 (83.4)	158 (83.3)
Missing	4 (0.2)	4 (0.2)	0 (0)
Tobacco use (N, %)			
Never	1290 (70.6)	1195 (73.1)	102 (49.5)
Current	312 (17.1)	257 (15.7)	58 (28.6)
Stopped	220 (12.0)	178 (10.9)	45 (22.0)
Missing	5 (0.5)	5 (0.3)	0 (0)
Presence of cough (N, %)			
Yes	1583 (86.6)	1385 (85.4)	186 (96.9)
No	245 (13.4)	239 (14.6)	6 (3.1)
Number of TB symptoms (N, %)			
No symptoms	87 (4.8)	87 (5.3)	0 (0)
One symptom	254 (13.9)	247 (15.1)	7 (3.6)
Two symptoms	404 (22.1)	368 (22.5)	36 (18.8)
Three or more symptoms	1082 (59.2)	933 (57.1)	149 (77.6)

* For the abnormality AI model, an additional 83 cases (*not listed here*) were used for evaluation (see “Methods”). IQR denotes interquartile range; and TB, tuberculosis.

## Data Availability

Data from this study, including de-identified CXR images, TB reference standard, and predictions from both AI models, will be made available (https://www.kaggle.com/datasets/googlehealthai/google-health-ai). Inquiries about access to the latest version of the AI models can be made to roryp@google.com.
