# Supplementary material for: Prospective Multi-Site Validation of AI to Detect Tuberculosis and Chest X-Ray Abnormalities
Source: NEJM AI. Author manuscript; Available in PMC 2025 Jan 16. (PMC11737584; doi:10.1056/aioa2400018)
Supplement: data sharing statement [file NIHMS2033608-supplement-data_sharing_statement.pdf]

Data Sharing Statement

Sahar Kazemzadeh, Atilla P. Kiraly, Zaid Nabulsi, et al. Prospective Multi-Site Validation of AI to Detect Tuberculosis and Chest X-Ray Abnormalities. NEJM Ai. DOI: 10.1056/AIoa2400018.

Question	Authors' Response
Will the data collected for your study be made available to others?	Yes
Would you like to offer context for your decision?	—
Which data?	—
Additional information about data	—
How or where can the data be obtained?	—
When will data availability begin?	—
When will data availability end?	—
Will any supporting documents be available?	—
Which supporting documents?	—
Additional information about supporting documents	—
How or where can supporting documents be obtained?	—
When will supporting documents availability begin?	—
When will supporting documents availability end?	—
To whom will data be available?	—
For what type of analysis or purpose?	—
By what mechanism?	—
Any other restrictions?	—
Additional information	—

This statement was posted on September 26, 2024, at ai.nejm.org.
