# Supplementary material for: Prospective Multi-Site Validation of AI to Detect Tuberculosis and Chest X-Ray Abnormalities
Source: NEJM AI. Author manuscript; Available in PMC 2025 Jan 16. (PMC11737584; doi:10.1056/aioa2400018)
Supplement: disclosures [file NIHMS2033608-supplement-disclosures.pdf]

Apr 24, 2024 01:48:50 EDT
NEJM AI

Arnav Agharwal

Discloser Identifier: 1156279

Disclosure Purpose: AI-24-00018.R1

Summary of Interests

Company or Organization

Entity	Type	Interest Held By
Google	Employment	Self
**Title:** Senior Software Engineer<br>**Additional Information:** Full-time employment.	**Position Description:**	
Google	Stock	Self
**Additional Information:** Stock offering as part of employment compensation strucuture.		

Certification

I certify that the information provided in this disclosure is complete and accurate.

Convey Global Disclosure System | AAMC

Christina Chen

Discloser Identifier: 1167388

Disclosure Purpose: AI-24-00018.R1

Summary of Interests

Company or Organization

Entity	Type	Interest Held By
Dexcom, Inc.	Employment	Self
**Title:** Senior Staff Clinician Scientist **Position Description:** Clinical Strategy **Additional Information:**		
Google	Stock	Self
**Additional Information:**		

Certification

I certify that the information provided in this disclosure is complete and accurate.

Convey Global Disclosure System AAMC

Katherine Chou

Discloser Identifier: 1153989

Disclosure Purpose: AI-24-00018.R1

Summary of Interests

I do not have any interests to disclose at this time.

Certification

I certify that the information provided in this disclosure is complete and accurate.

Convey Global Disclosure System | AAMC

Sep 10, 2024 04:08:59 EDT
NEJM AI

Greg Corrado

Discloser Identifier: 1152293

Disclosure Purpose: AI-24-00018.R1

Summary of Interests

Company or Organization

Entity	Type	Interest Held By
Google	Employment	Self
**Title:** Distinguished Scientist<br>**Additional Information:**	**Position Description:**	
Google	Stock	Self
**Additional Information:**		

Intellectual Property

Type	Is Licensed	Interest Held By
Patent - Multiple AI/ML patents.	No	Self
**Description:** Multiple AI/ML patents.<br>**Patent Status:**<br>**Filing Jurisdiction:** USA<br>**Licensees:**<br>**Additional Information:**	**Patent Number:**<br>**Patent Holder:** Current Institution	

Certification

I certify that the information provided in this disclosure is complete and accurate.

Convey Global Disclosure System AAMC

Apr 02, 2024 03:07:54 EDT
NEJM AI

Krishnan Eswaran

Discloser Identifier: 1153995

Disclosure Purpose: AI-24-00018.R1

Summary of Interests

Company or Organization

Entity	Type	Interest Held By
Google	Employment	Self

Title: Senior Staff Software Engineer
Position Description:
Additional Information: In addition to compensation, this also includes stock / equity in the company

Certification

I certify that the information provided in this disclosure is complete and accurate.

Convey Global Disclosure System AAMC

Apr 05, 2024 11:12:45 EDT
NEJM AI

Daniel Golden

Discloser Identifier: 1153993

Disclosure Purpose: AI-24-00018.R1

Summary of Interests

Company or Organization

Entity	Type	Interest Held By
Google	Employment	Self

Title: Software Engineer
Position Description:
Additional Information:

Certification

I certify that the information provided in this disclosure is complete and accurate.

Convey Global Disclosure System AAMC

Shahar Jamshy

Discloser Identifier: 1167387

Disclosure Purpose: AI-24-00018.R1

Summary of Interests

Company or Organization

Entity	Type	Interest Held By
Google	Employment	Self
**Title:** Software Engineer<br>**Additional Information:**	**Position Description:** Worked with Google Health AI group	
Google	Stock	Self
**Additional Information:**		

Certification

I certify that the information provided in this disclosure is complete and accurate.

Convey Global Disclosure System | AAMC

Apr 02, 2024 05:51:11 EDT
NEJM AI

Sahar Kazemzadeh

Discloser Identifier: 1153977

Disclosure Purpose: AI-24-00018.R1

Summary of Interests

Company or Organization

Entity	Type	Interest Held By
Google	Employment	Self
**Title:** Software Engineer<br>**Additional Information:**	**Position Description:**	

Certification

I certify that the information provided in this disclosure is complete and accurate.

Convey Global Disclosure System | AAMC

Atilla Kiraly

Discloser Identifier: 1153976

Disclosure Purpose: AI-24-00018.R1

Summary of Interests

Company or Organization

Entity	Type	Interest Held By
Google	Employment	Self

Title: SWE
Position Description: SWE
Additional Information: Includes stock and associated patents

Certification

I certify that the information provided in this disclosure is complete and accurate.

Convey Global Disclosure System AAMC

Apr 26, 2024 11:33:44 EDT
NEJM AI

Charles Lau

Discloser Identifier: 1152183

Disclosure Purpose: AI-24-00018.R1

Summary of Interests

Company or Organization

Entity	Type	Interest Held By
Google	Consultant	Self
**Category:** Consultant<br>**Description:** Radiologist Consultant<br>**Additional Information:**		

Certification

I certify that the information provided in this disclosure is complete and accurate.

Convey Global Disclosure System | AAMC

Yun Liu

Discloser Identifier: 1148170

Disclosure Purpose: AI-24-00018.R1

Summary of Interests

Company or Organization

Entity	Type	Interest Held By
Google	Employment	Self

Title: Senior Staff Research Scientist
Additional Information:
Position Description: Research lead for multiple AI projects in healthcare

Entity	Type	Interest Held By
Google	Stock	Self

Additional Information: Alphabet stock ownership

Intellectual Property

Type	Is Licensed	Interest Held By
Patent - Determining Chest Conditions from Radiograph Data	No	Self

Description: Determining Chest Conditions from Radiograph Data via Machine Learning
Patent Status: Pending
Filing Jurisdiction: USA
Licensees:
Additional Information:
Patent Number: US20230169652A1
Patent Holder: Current Institution

Certification

I certify that the information provided in this disclosure is complete and accurate.

Convey Global Disclosure System | AAMC

Apr 18, 2024 04:52:39 EDT
NEJM AI

Minyoi Maimbolwa

Discloser Identifier: 1167385

Disclosure Purpose: AI-24-00018.R1

Summary of Interests

I do not have any interests to disclose at this time.

Certification

I certify that the information provided in this disclosure is complete and accurate.

Convey
Global Disclosure System
AAMC

Yossi Matias

Discloser Identifier: 1152294

Disclosure Purpose: AI-24-00018.R1

Summary of Interests

I do not have any interests to disclose at this time.

Certification

I certify that the information provided in this disclosure is complete and accurate.

Convey Global Disclosure System | AAMC

Monde Muyoyeta

Discloser Identifier: 1167392

Disclosure Purpose: AI-24-00018.R1

Summary of Interests

I do not have any interests to disclose at this time.

Certification

I certify that the information provided in this disclosure is complete and accurate.

Convey Global Disclosure System | AAMC

Zaid Nabulsi

Discloser Identifier: 1167382

Disclosure Purpose: AI-24-00018.R1

Summary of Interests

Company or Organization

Entity	Type	Interest Held By
Google	Employment	Self
**Title:** Software Engineer<br>**Additional Information:**	**Position Description:**	
Google	Stock	Self
**Additional Information:**		

Certification

I certify that the information provided in this disclosure is complete and accurate.

Rory Pilgrim

Discloser Identifier: 1153994

Disclosure Purpose: AI-24-00018.R1

Summary of Interests

Company or Organization

Entity	Type	Interest Held By
Google	Employment	Self
**Title:** Product Manager **Additional Information:**	**Position Description:** Manage Products	
Google	Stock	Self
**Additional Information:**		

Certification

I certify that the information provided in this disclosure is complete and accurate.

Convey Global Disclosure System | AAMC

Shruthi Prabhakara

Discloser Identifier: 1153992

Disclosure Purpose: AI-24-00018.R1

Summary of Interests

I do not have any interests to disclose at this time.

Certification

I certify that the information provided in this disclosure is complete and accurate.

Convey Global Disclosure System | AAMC

NSALA Sanjase

Discloser Identifier: 1167384

Disclosure Purpose: AI-24-00018.R1

Summary of Interests

I do not have any interests to disclose at this time.

Certification

I certify that the information provided in this disclosure is complete and accurate.

Convey Global Disclosure System | AAMC

Andrew Sellergren

Discloser Identifier: 1150638

Disclosure Purpose: AI-24-00018.R1

Summary of Interests

Company or Organization

Entity	Type	Interest Held By
Google	Employment	Self

Title: Software Engineer

Position Description: Research, development, and deployment of models for Health AI

Additional Information: salary, stock plan

Certification

I certify that the information provided in this disclosure is complete and accurate.

Convey Global Disclosure System AAMC

Shravya Shetty

Discloser Identifier: 1153990

Disclosure Purpose: AI-24-00018.R1

Summary of Interests

Company or Organization

Entity	Type	Interest Held By
Google	Stock	Self
**Additional Information:**		

Certification

I certify that the information provided in this disclosure is complete and accurate.

Convey Global Disclosure System | AAMC

Apr 16, 2024 04:34:12 EDT
NEJM AI

Brian Shuma

Discloser Identifier: 1167386

Disclosure Purpose: AI-24-00018.R1

Summary of Interests

I do not have any interests to disclose at this time.

Certification

I certify that the information provided in this disclosure is complete and accurate.

Convey Global Disclosure System | AAMC

Apr 04, 2024 12:30:25 EDT
NEJM AI

Daniel Tse

Discloser Identifier: 1153991

Disclosure Purpose: AI-24-00018.R1

Summary of Interests

Company or Organization

Entity	Type	Interest Held By
Google	Employment	Self

Title: Product Manager **Position Description:**
Additional Information: Salary + company stock as part of normal stock compensation package. Also named in patents held by Google listed here: https://patents.google.com/?q=(google)&inventor=daniel+tse&oq=daniel+tse+google&sort=new

Certification

I certify that the information provided in this disclosure is complete and accurate.

Convey Global Disclosure System AAMC

Apr 12, 2024 04:35:11 EDT
NEJM AI

Eric Wu

Discloser Identifier: 1167391

Disclosure Purpose: AI-24-00018.R1

Summary of Interests

I do not have any interests to disclose at this time.

Certification

I certify that the information provided in this disclosure is complete and accurate.

Convey Global Disclosure System | AAMC

Dong Jin Yu

Discloser Identifier: 1167390

Disclosure Purpose: AI-24-00018.R1

Summary of Interests

Company or Organization

Entity	Type	Interest Held By
Google	Employment	Self
**Title:** Software Engineer **Additional Information:** I was a Google employee and owned Alphabet stock	**Position Description:**	
Google	Stock	Self
**Additional Information:** I was a Google employee and owned Alphabet stock		

Certification

I certify that the information provided in this disclosure is complete and accurate.

Convey Global Disclosure System | AAMC
