# Supplementary material for: Prospective Multi-Site Validation of AI to Detect Tuberculosis and Chest X-Ray Abnormalities
Source: NEJM AI. Author manuscript; Available in PMC 2025 Jan 16. (PMC11737584; doi:10.1056/aioa2400018)
Supplement: appendix [file NIHMS2033608-supplement-appendix.pdf]

Supplementary Appendix

This appendix has been provided by the authors to give readers additional information about their work.

Sahar Kazemzadeh, Atilla P. Kiraly, Zaid Nabulsi, et al. Prospective Multi-Site Validation of AI to Detect Tuberculosis and Chest X-Ray Abnormalities. NEJM Ai. DOI: 10.1056/AIoa2400018.

Supplementary Information

Supplementary Methods

Data collection and data management

Data were collected using paper case record forms administered by the study clinical teams. No identifying data was collected; study participants were assigned a unique study number. This study number was used to capture data into a password-controlled electronic database. Access was only available to study teams. Electronic data underwent quality checks and data verification before data lock.

The database was developed on the Digital Health Information Software 2 (DHIS-2) platform. Inbuilt features of the DHIS-2 application were utilized to ensure access control was maintained. Users were granted access at organization unit or health facility level to ensure that users were only able to capture and access data at the specific site where they operate. Furthermore, access control was applied at CRF level, where users were only granted access to capture, update or edit CRFs which were applicable to them.

The database was implemented as a tracker application, with the different CRFs represented as different stages in the tracker application.

Data validation checks were carried out every week and involved the sharing of validation reports on extracted data highlighting and giving feedback to the data teams to update and reconcile data flagged in one or more of the following categories:

1. Incomplete screening and enrollments
2. Incomplete symptoms and clinic history
3. Missing chest X-ray
4. Missing baseline sputum collection
5. Missing baseline sputum quality
6. Missing baseline smear results
7. Missing baseline Xpert Ultra results
8. Missing baseline MTB culture results
9. Missing TB diagnosis
10. Month 1 follow-ups due for clinically diagnosed TB patients
11. Month 1 follow-ups due for bacteriologically confirmed TB patients
12. Month 2 follow-ups due for clinically diagnosed TB patients
13. Month 2 follow-ups due for bacteriologically confirmed TB patients

Once the data was updated and reconciled it was excluded from subsequent reports in the following weeks. This process was continuous throughout the duration of the study, ultimately with the aim of eventually ending up with zero output in the validation reports.

After a protocol amendment during the study, approximately 600 participants additionally had cough audio recorded; the audio data was collected for planned future work and was not used as part of this CXR AI validation.

TB and Abnormality AI model details

Both AI models were updated for the study from previous publications. This involved retraining and as well as the introduction of new architectures.

The TB AI used the same EfficientNet B7 architecture as in the previous publication[6], but was retrained. Test sets from the previous publication were incorporated as training data, cross entropy loss was replaced with focal loss, and the model was pre-trained on an improved abnormality AI. The system was simplified to remove lung cropping from the preprocessing without degradation to eval metrics. Finally, the eval metric was updated to partial AUC over the 80-95% sensitivity range to emphasize model improvements over the clinically relevant portion of the AUC curve.

The abnormality AI model was updated from a single model[13] to an ensemble of six and the architecture changed from an EfficientNet B7 to ResNet-based architectures along with higher resolution input images. Contrastive learning was used to train the updated architectures. As a result, in comparison to performance on two previous datasets, DS-1 and CXR-14, the new AI model achieved AUCs of 0.892 versus 0.870 and 0.969 versus 0.940, respectively.

AI model threshold selection process

Each of the TB and abnormality AIs produced a score between 0 and 1, which were subsequently thresholded to get binary “yes/no” outputs. Thresholds for both AI models were pre-selected using a pilot phase of 493 patients preceding the main study alongside datasets used in the respective published retrospective studies (Nabulsi et al., Nature Scientific Reports, 2021; Kazemzadeh and Yu et al., Radiology, 2022;). None of the threshold-selection datasets were used for statistical analysis presented in this manuscript.

The threshold selection process involved plotting the estimated statistical power using the projected sample size in the formal (non-pilot) phase of the study for various thresholds for each of the models, and using different reference standard definitions to understand sensitivity to the ground truth. Specifically, the respective datasets described in the previous paragraph and number of radiologists were upsampled to the projected size in the formal study, and the prespecified statistical tests were conducted. Bootstrapping the datasets and repeating the tests to estimate the frequency of rejecting the null hypothesis at each threshold results in a plot of statistical power (1 line for sensitivity power, 1 line for specificity power, on the y-axis) against the threshold on the x-axis. There is a trade off: as the threshold increases from 0, the number of predicted positives decreases, so sensitivity decreases from 100%, and specificity increases from 0%. As such, statistical power decreases for sensitivity and increases for specificity. These opposite trends result in a middle area where statistical power is most optimal for both tests.

For the TB AI model, the high sensitivity threshold was set at 0.305, estimated to have at least 0.9 power to achieve non-inferiority vs. radiologists for sensitivity and non-inferiority vs. the WHO targets (90% sensitivity and 70% specificity) for both sensitivity and specificity. The exploratory, more balanced threshold was set at 0.465, with at least 0.6 power for non-inferiority vs. radiologists for both sensitivity and specificity. Given the WHO goals of favoring higher sensitivity over specificity, the high sensitivity threshold was selected as the primary analysis.

The abnormality AI model was projected to achieve the pre-specified 90% sensitivity and 50% specificity targets (set to optimize for high sensitivity) with near perfect power across a range of thresholds. As such, in performing power estimation for threshold optimization purposes, a more stringent 95% sensitivity and 80% specificity was used. The thresholds of 0.54 and 0.67 spanned the region yielding high statistical power (exceeding approximately 0.9), and were selected. The higher sensitivity threshold of 0.54 was selected as the main analysis, again to favor sensitivity over specificity given the importance of minimizing false negatives.

The TB AI's scores were dichotomized into labels of "Active TB suspected" and "Active TB not suspected". The abnormality AI's scores were dichotomized into labels of "Actionable findings suspected" and "Actionable findings not suspected." The abnormality AI did not output scores for specific findings, partially because many findings would have been too rare to evaluate reliably, without careful enrichment. The dichotomized labels were printed onto the CXR, and the raw scores and binarized labels were inserted into private DICOM tags.

Supplementary Results

TB AI

Supplementary Tables 1-3 analyze the TB AI performance while matching its sensitivity and specificity to that of individual radiologists as well as average radiologist performance and WHO targets. Supplementary Figures 1-4 (panel A in each figure) present the TB AI results on subgroups based on previous TB status, sex, age, and site, respectively.

Supplementary Figure 5 and Supplementary Table 4 presents subgroups based on smear status (all smear positive patients were also TB positive, so instead of a ROC curve, sensitivity is presented). Supplementary Figure 6 presents the positive flagging rate for every radiologist. Supplementary Figure 11 presents the subgroups based on radiologist-indicated image quality. Due to small numbers of images labeled with presence vs absence of image quality concerns, it is challenging to interpret the image of image quality.

Visualizations of the selected thresholds relative to reader responses is shown in Supplementary Figure 7. The histogram of AI outputs shows a distribution of scores with a broad peak near 0.1 and a tail that extends to 0.9. At the selected thresholds, at least 20% to 50% of the radiologists identified the cases as positive, demonstrating that the thresholds were more sensitive than most readers.

Abnormality AI

Supplementary Figures 1-4 (panel B in each figure) present the abnormality AI results on subgroups based on previous TB status, sex, age, and site, respectively. The high-sensitivity threshold selected

achieved over 90% sensitivity and 50% specificity in all subgroups except for ages > 65 where sensitivity was 100% and specificity was 29.6%.

Visualizations of the selected thresholds relative to reader responses is shown in Supplementary Figure 8. The histogram of AI outputs shows a distribution of scores with a narrow peak at 1 and a broader peak in the 0-0.2 range. At the selected thresholds, at least 30% to 40% of the radiologists identified the cases as positive, demonstrating that the thresholds were more sensitive than most readers. Supplementary Figure 9 is a sensitivity analysis, recomputing the abnormality AI's AUC based on all possible combinations of 3 radiologists from the pool of 9 who read the cases for abnormalities. The median AUC from these combinations was greater than that of the ground truth used.

Age subgroup analysis

To dive into unanticipated differences in subgroup performance, we conducted further exploratory analysis. First, we performed multivariable analysis using a logistic regression model to predict AI sensitivity (or specificity), and added as predictors demographic factors. Multivariable analysis was done for both the TB AI and the abnormality AI, and for both sensitivity and specificity. Performance differences from the subgroup analyses were also observed in this analysis, including differences with HIV, previous TB, sex, and age. We observed that age was correlated with HIV and previous TB (which were individually associated with lower specificity), and thus stratified the age subgroup analysis to patients without HIV and no prior TB (Supplementary Figure 12). The age-associated trends towards lower performance for both AI and radiologists recovered, indicating that these 2 factors were drivers of this age trend.

Examination of model results on edge cases

Cases where the TB AI model and abnormality AI disagreed and readers disagreed were characterized by a US-based board certified radiologist with 19 years of experience. The radiologist was given both model outputs, HIV status, and if the case had disagreements by the TB radiologists (i.e. half of the readers scored the case as TB positive). A total of 24 cases were examined.

In TB positive cases where the TB AI marked the case negative, many contained only faint/subtle focal lung opacities and in some cases, scarring. In TB negative cases marked positive by the TB AI, cases were mostly normal, but contained artifacts of one type or another that resulted in appearances that can mimic lung opacities. These artifacts included asymmetric overlying breast tissue, respiratory motion blur, and obesity/large body habitus.

Cases where the TB AI model correctly called a positive or negative but the abnormality AI made the opposite call were also analyzed. Among the TB AI true positives were the abnormality AI marked negative, roughly half had a mass-like lung opacity and one had a diffuse micronodular interstitial pattern. Finally, within the TB AI true negative cases where the abnormality AI was positive, a variety of findings included faint focal patchy lung opacities, focal infectious bronchiolitis pattern, solitary lung nodule, diffuse image noise due to obese patient/large body habitus mimicking diffuse heterogeneous lung opacities, and lung hyperinflation/COPD.

Among the cases read, a possible defect in the detector was identified, as shown in Supplementary Figure 10.

Supplementary Figures

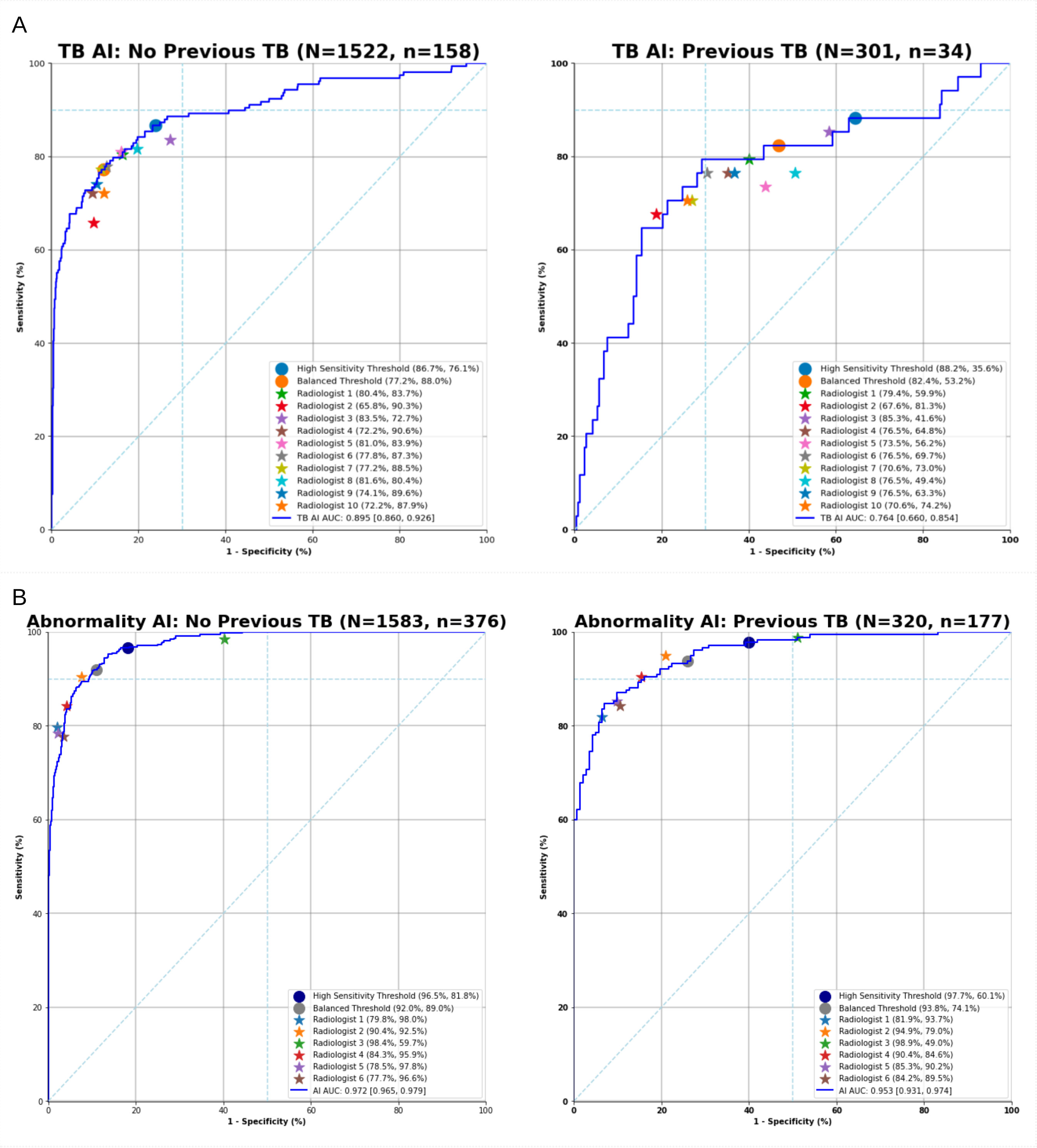

Supplementary Figure 1: Subgroup analysis by previous TB for the (A) TB AI and (B) abnormality AI.

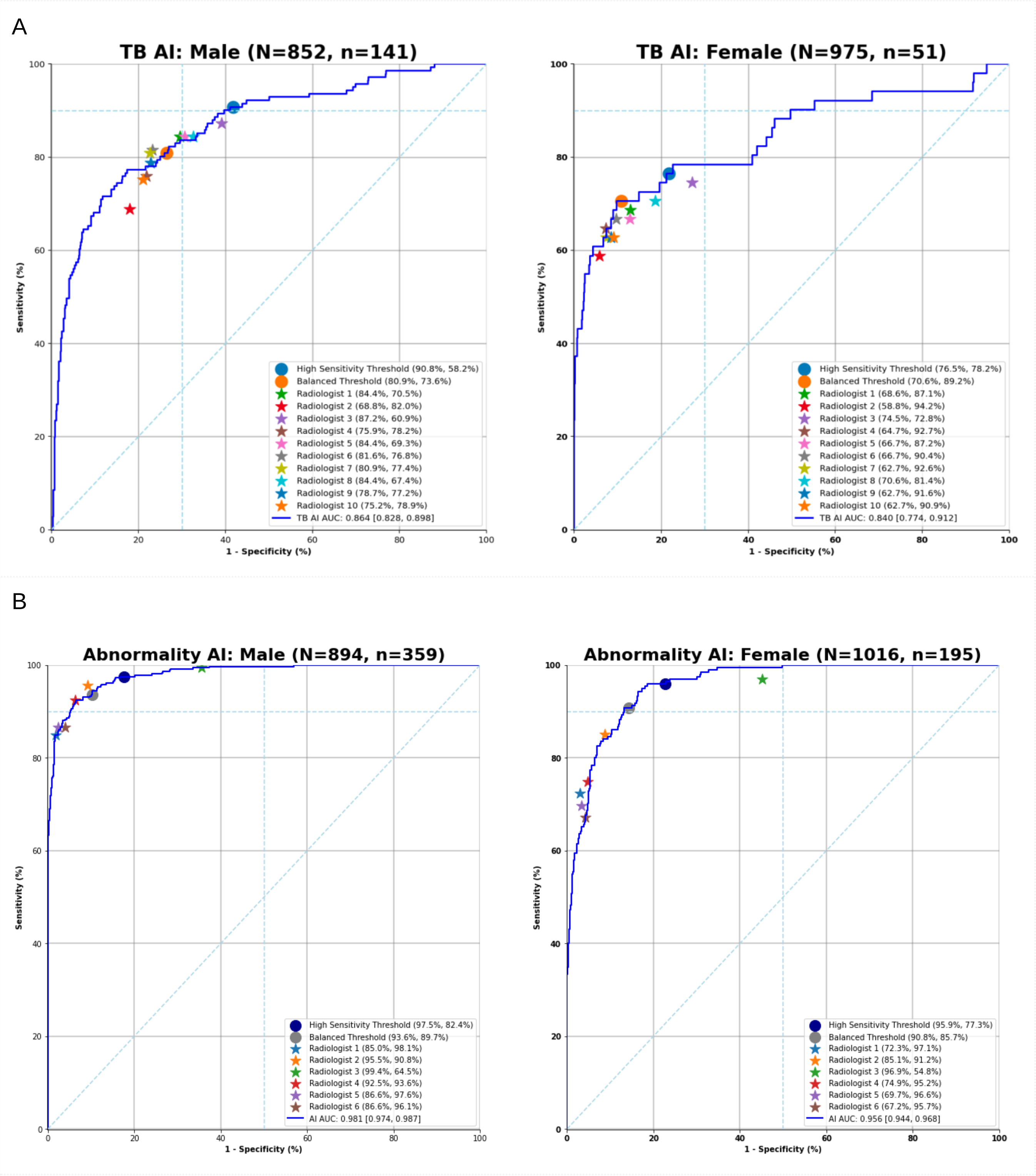

Supplementary Figure 2: Subgroup analysis by sex for the (A) TB AI and (B) abnormality AI.

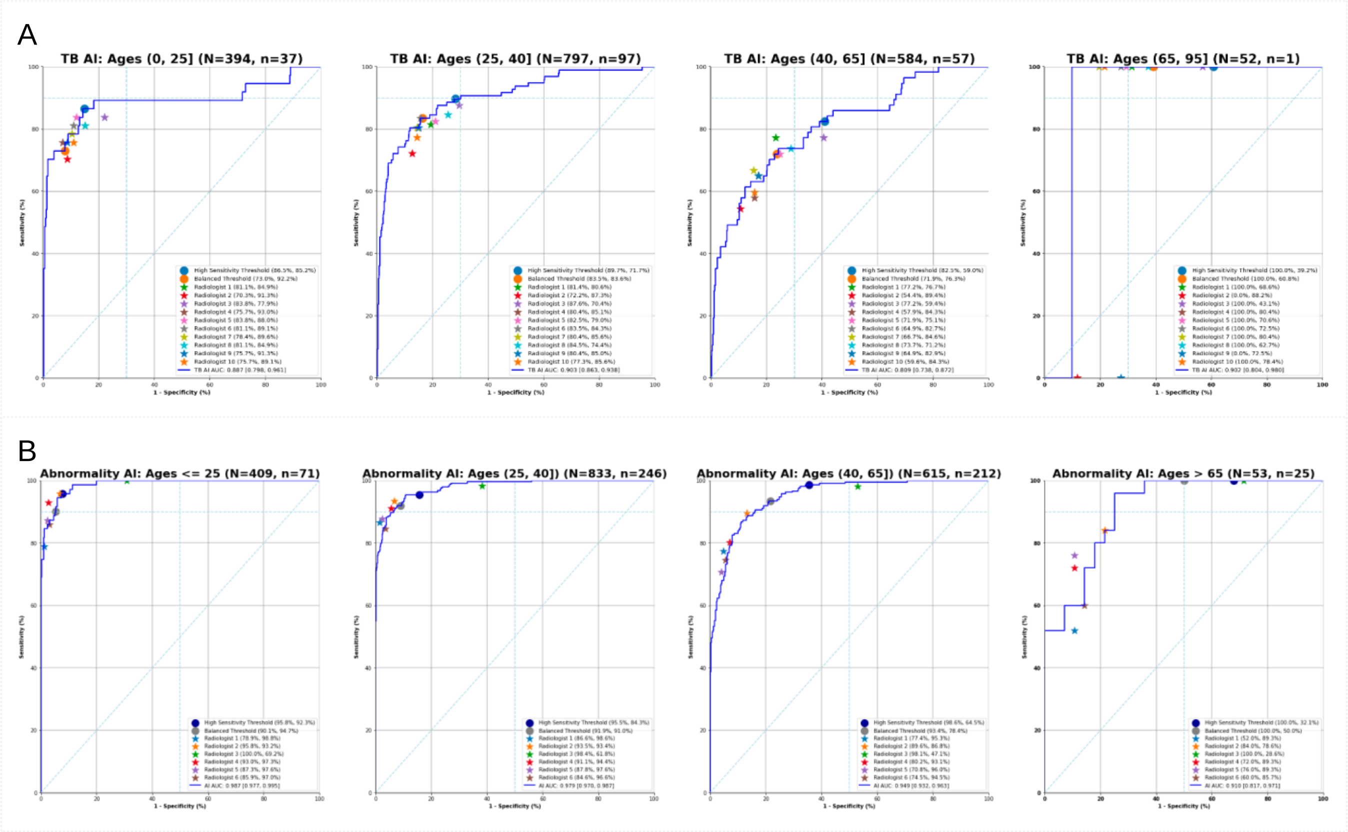

Supplementary Figure 3: Subgroup analysis by age for the (A) TB AI and (B) abnormality AI.

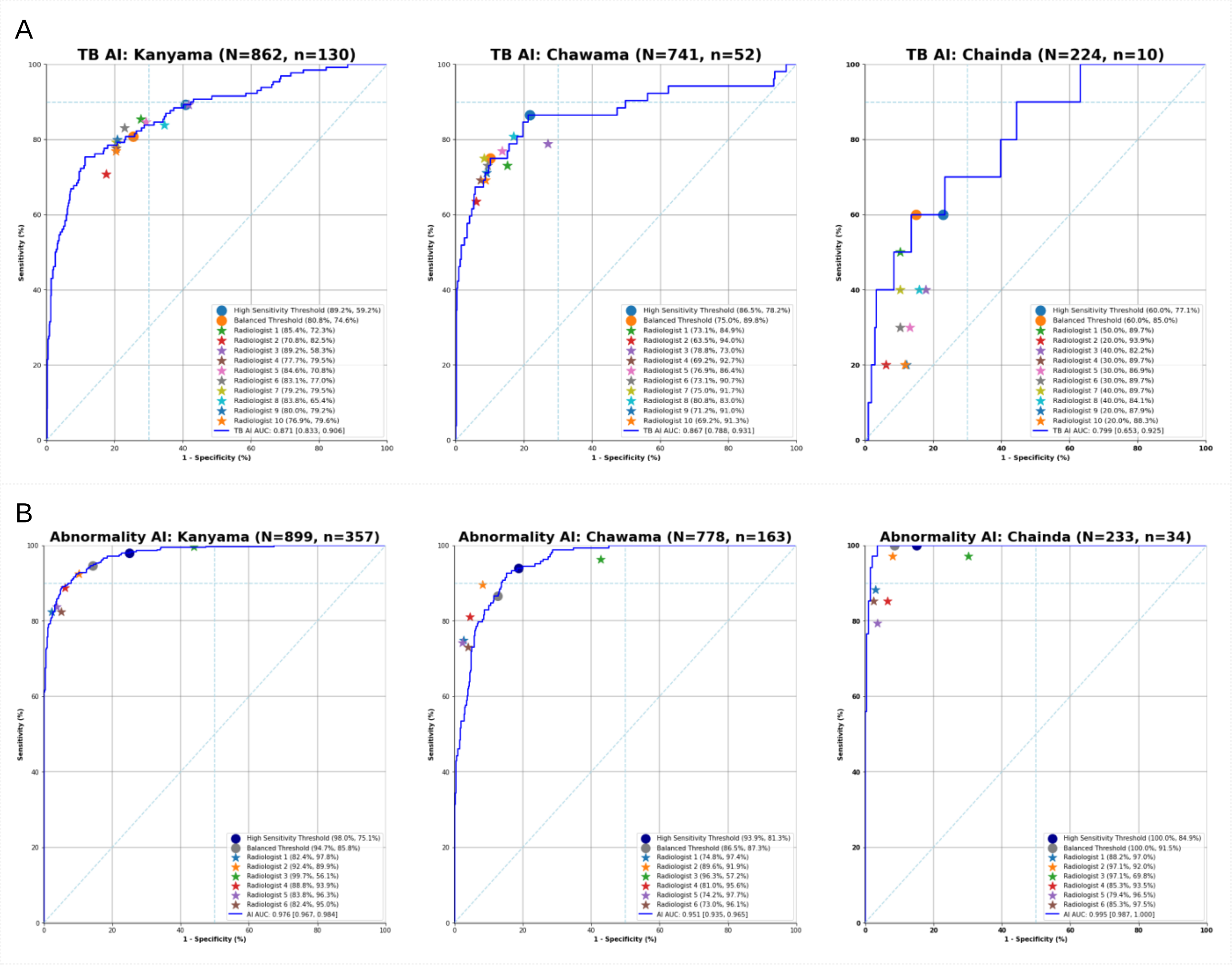

Supplementary Figure 4: Subgroup analysis by site for the (A) TB AI and (B) abnormality AI.

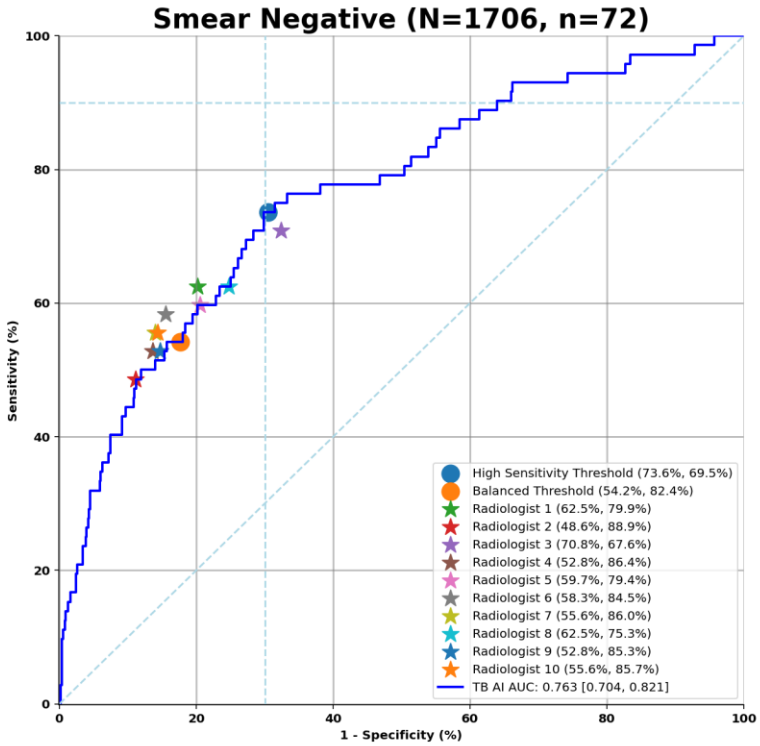

Supplementary Figure 5: TB AI performance for smear negative patients

Because all smear positive cases were also TB positive, an ROC curve (which requires both positives and negative examples) could not be plotted; instead we present the sensitivity of the TB AI and the radiologists in Supplementary Table 4.

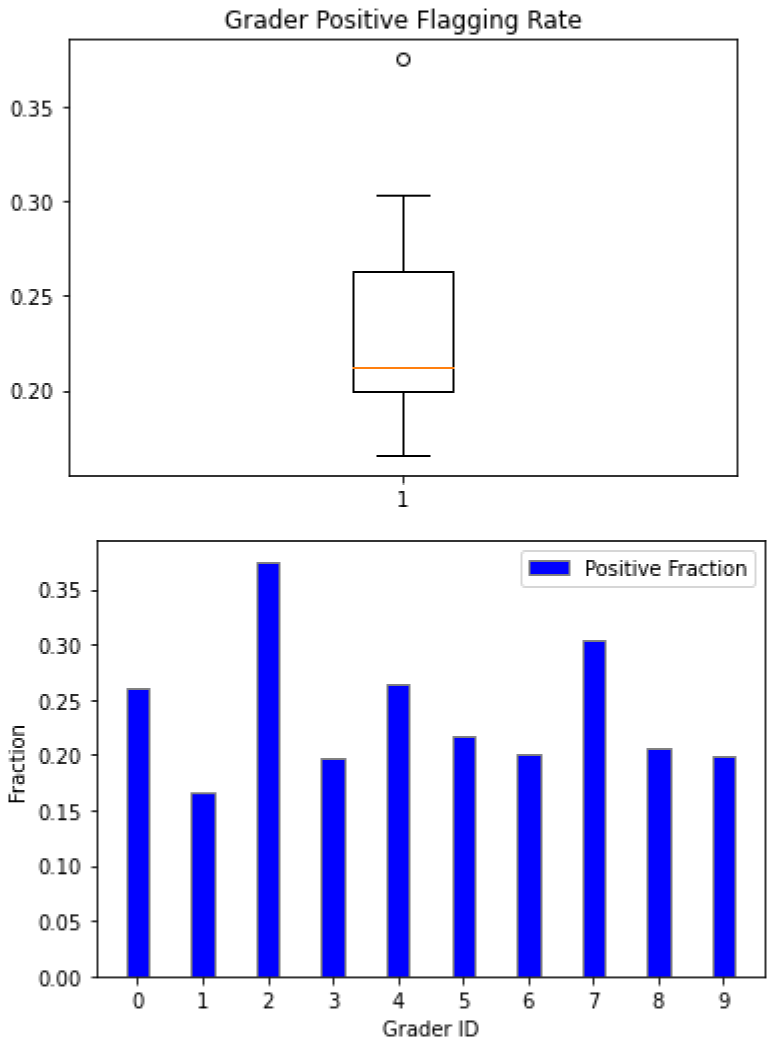

Supplementary Figure 6: Positive flagging rate for TB radiologists.

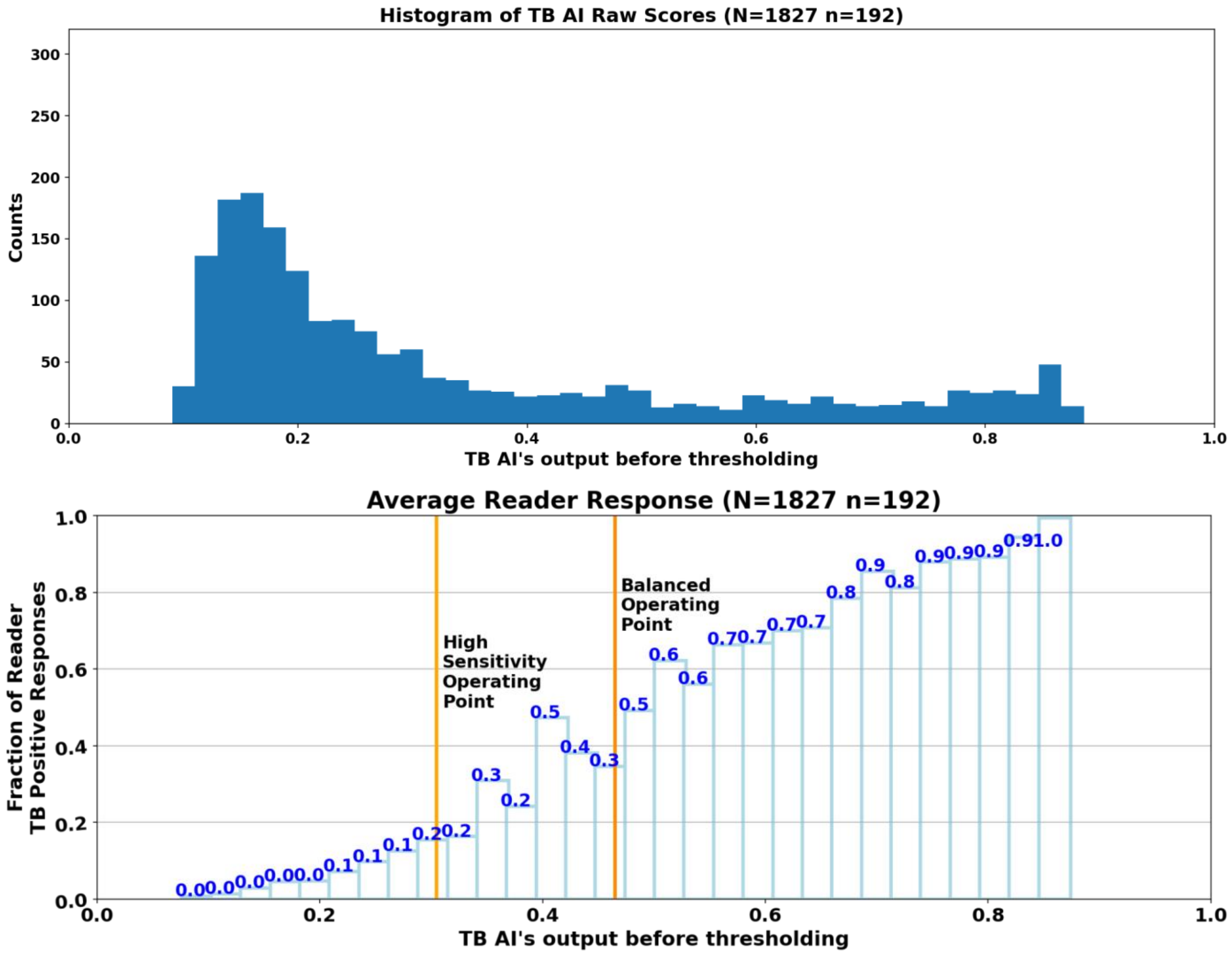

Supplementary Figure 7: Distribution of the TB AI predictions in comparison with radiologists. (Top) Histogram of the TB AI’s continuous predictions (before thresholding). (Bottom) The average reader response from all 10 comparator radiologists who reviewed for TB status. The two orange lines show the selected study thresholds (operating points). The lower plot is the average of the 10 radiologists indicating the presence of TB per bucket of cases. Overall, the rates of the radiologists indicating the presence of TB increased with the AI’s predictions, and the two thresholds were more sensitive than most of the readers.

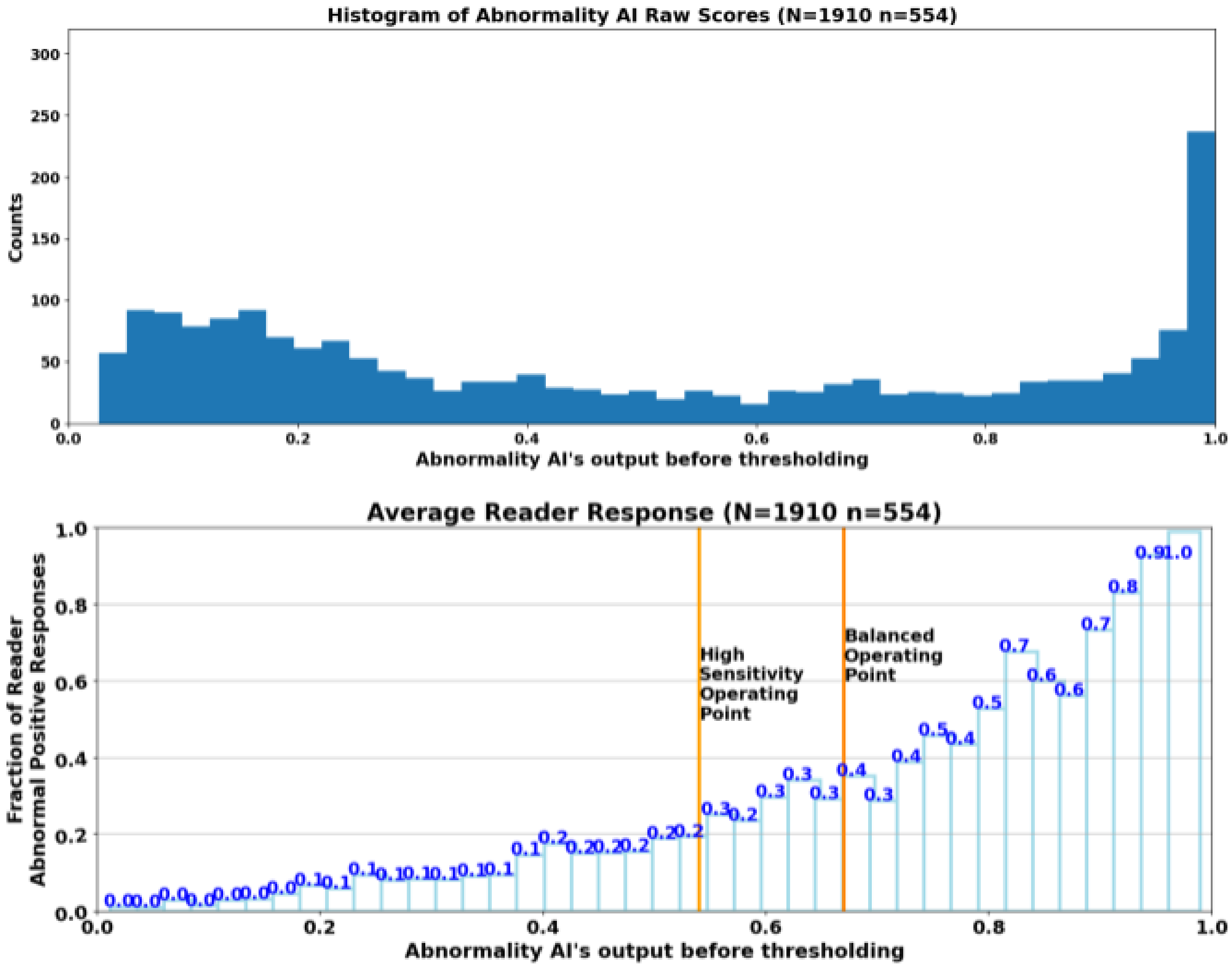

Supplementary Figure 8: Distribution of the abnormality AI predictions in comparison with radiologists. (Top) Histogram of the abnormality AI's continuous predictions (before thresholding). (Bottom) Using the same bucketing, the average reader response from all 9 radiologists who reviewed for CXR abnormalities is plotted. The two orange lines show the selected study thresholds (operating points). The lower plot is the average of the 9 radiologists indicating the presence of abnormal CXR findings per bucket of cases. Overall, the rates of the radiologists indicating the presence of abnormal findings increased with the AI's predictions, and the two thresholds were more sensitive than most of the readers.

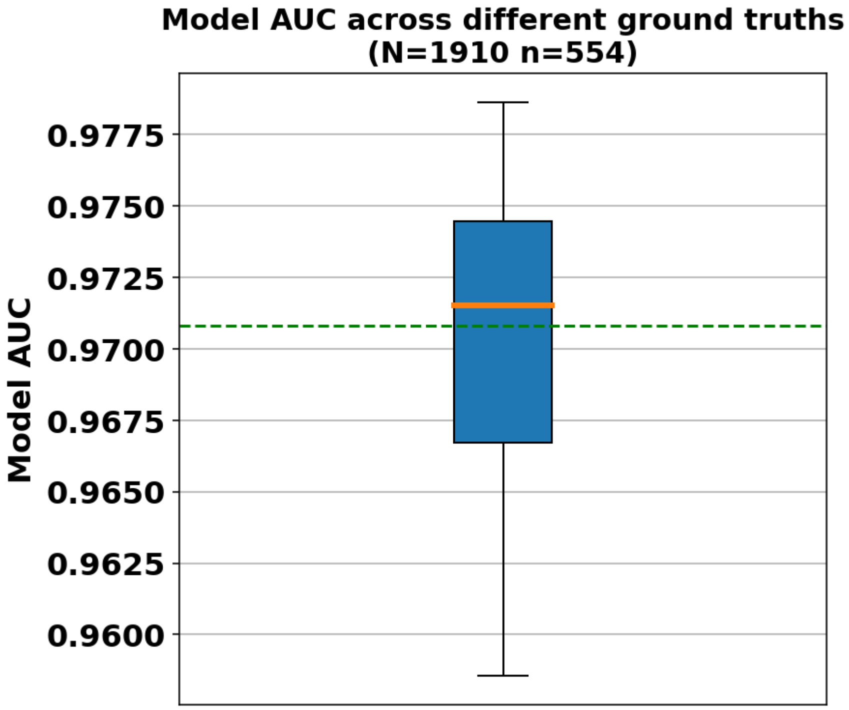

Supplementary Figure 9: Sensitivity analysis for the choice of the 3 radiologists comprising the reference standard for the abnormality AI. The box-plot indicates the abnormality AI's AUCs computed against all combinations of 3 radiologist readers from the overall pool of 9, instead of the 3 experienced readers (green dashed line).

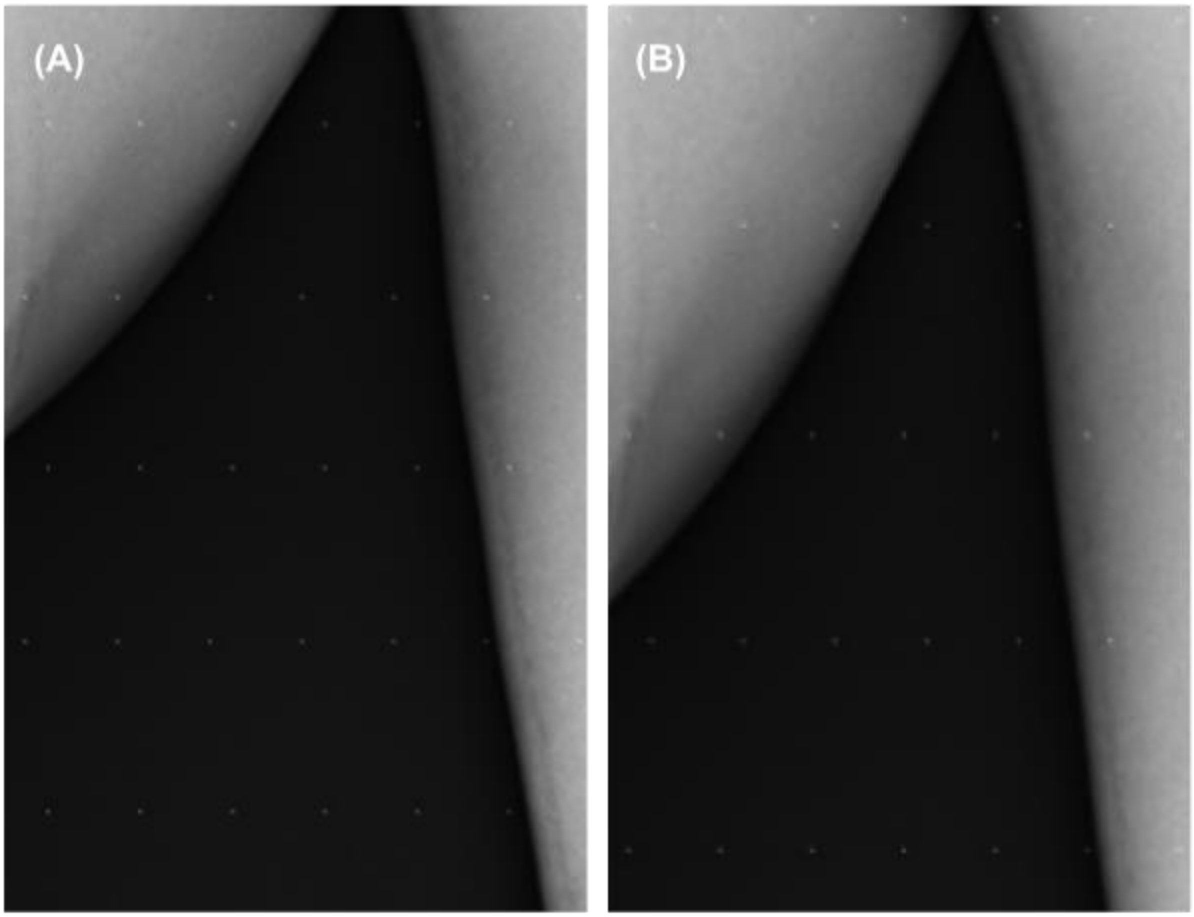

Supplementary Figure 10: Grid-like artifacts, possibly due a damaged detector. These were present on all images from Kanyama and Chawama due to a shared detector. (A) presents this artifact in a close-up view of the original image and resolution, while (B) visualizes the artifact on the same section after preprocessing and downsampling (i.e., what the AI model saw). Both images are resized to be approximately the same size for display purposes. Artifacts become blurred in the processed image.

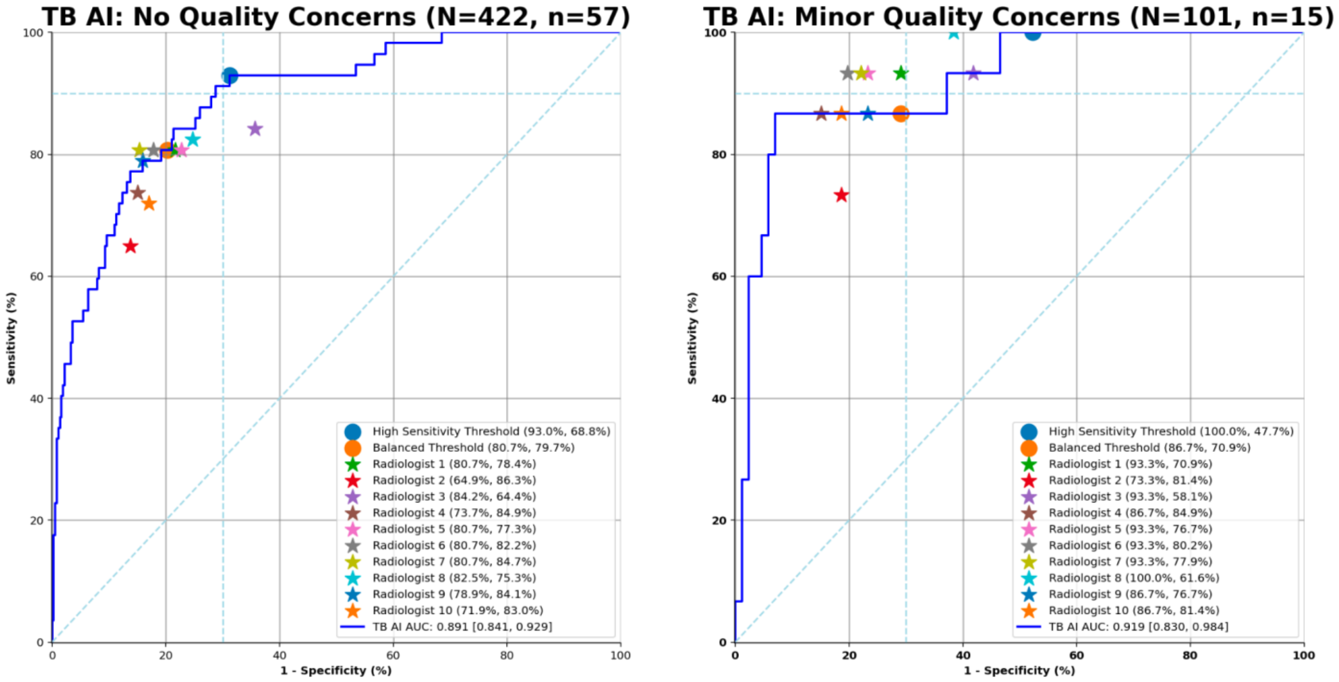

Supplementary Figure 11: Subgroup analysis by radiologist-indicated minor image quality concerns for TB AI.

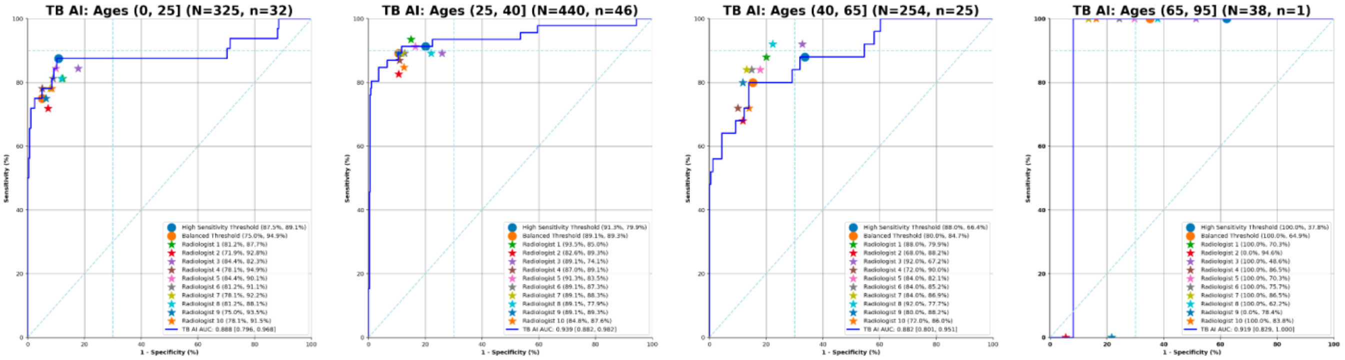

Supplementary Figure 12: Subgroup analysis by age for HIV-negative, previous TB negative patients for TB AI.

Supplementary Tables

Radiologist	Radiologist Sensitivity	TB AI Sensitivity	Radiologist Specificity	TB AI Specificity	Delta
1	77.6%	77.6%	84.5%	83.6%	-0.9%
2	76.0%	76.0%	86.0%	84.3%	-1.7%
3	80.2%	80.2%	79.9%	79.8%	-0.1%
4	74.5%	74.5%	85.3%	86.0%	0.7%
5	79.7%	79.7%	79.4%	80.6%	1.2%
6	72.9%	72.9%	86.4%	88.8%	2.4%
7	71.9%	71.9%	85.7%	89.1%	3.4%
8	80.7%	80.2%	75.3%	79.8%	4.5%
9	66.1%	66.7%	88.9%	92.8%	3.9%
10	83.9%	83.9%	67.6%	73.9%	6.3%
Average	76.4%	76.4%	81.9%	83.9%	2.0%

Supplementary Table 1: TB AI specificity at matched radiologist sensitivity. The last row is the average of those above and does not represent the performance of the matched to the average radiologist.

Radiologist	Radiologist Specificity	TB AI Specificity	Radiologist Sensitivity	TB AI Sensitivity	Delta
1	84.5%	84.3%	77.6%	76.0%	-1.6%
2	86.0%	86.0%	76.0%	74.5%	-1.5%
3	79.9%	79.8%	80.2%	80.2%	0.0%
4	85.3%	85.3%	74.5%	75.0%	0.5%
5	79.4%	79.8%	79.7%	80.2%	0.5%
6	86.4%	86.0%	72.9%	74.5%	1.6%
7	85.7%	86.0%	71.9%	74.5%	2.6%
8	75.3%	75.2%	80.7%	82.3%	1.6%
9	88.9%	88.8%	66.1%	72.9%	6.8%
10	67.6%	67.5%	83.9%	88.0%	4.1%
Average	81.9%	81.9%	76.4%	77.8%	1.5%

Supplementary Table 2: TB AI sensitivity at matched radiologist specificity. The last row is the average of those above and does not represent the performance of the matched to the average radiologist.

TB AI matched to:	TB AI sensitivity	TB AI specificity	Delta compared to radiologist performance
Average radiologist sensitivity (76.30%)	76.60%	84.00%	2.10% higher specificity
Average radiologist specificity (81.90%)	78.60%	82.00%	2.30% higher sensitivity
WHO target sensitivity (90%)	90.10%	49.60%	N/A
WHO target specificity (70%)	87.00%	70.20%	N/A

Supplementary Table 3: TB AI performance matched to specific targets. These analyses are based on posthoc matching of the TB AI model outputs to observed radiologist performances (both of which require inferring the TB AI's sensitivity and specificity characteristics for a range of thresholds, and thus requires the reference TB status from cultures).

Grader	Sensitivity
High Sensitivity Threshold	95.0%
Balanced Threshold	92.5%
Radiologist Average	87.4%
Radiologist 1	90.8%
Radiologist 2	76.7%
Radiologist 3	91.7%
Radiologist 4	85.0%
Radiologist 5	91.7%
Radiologist 6	89.2%
Radiologist 7	88.3%
Radiologist 8	91.7%
Radiologist 9	87.5%
Radiologist 10	81.7%

Supplementary Table 4: TB AI and radiologist sensitivity on smear positive patients (n=120).
